# Mechanism and Prediction of Gray Jujube Fruit Quality Using Explainable ANN

**DOI:** 10.1002/fsn3.70928

**Published:** 2025-09-16

**Authors:** Mingyang Yu, Yang Li, Junkai Zeng, Weifan Fan, Lanfei Wang, Hao Wang, Jiaxin Li, Jianping Bao

**Affiliations:** ^1^ Tarim Basin Biological Resources Protection and Utilization Key Laboratory, Xinjiang Production and Construction Corps Alar China; ^2^ Southern Xinjiang Special Fruit Trees High‐Quality High‐Quality Cultivation and Deep Processing of Fruit Products Processing Technical National Local Joint Engineering Laboratory Alar China; ^3^ College of Horticulture and Forestry Science Nanjing Agricultural University Nanjing China

**Keywords:** artificial neural network, bayesian optimization, gray jujube, quality prediction, SHAP analysis

## Abstract

Gray jujube ( *Ziziphus jujuba* Mill) is an important economic fruit crop in Xinjiang, China, whose fruit quality is regulated by complex interactions among tree architecture, physiological functions, and environmental factors. Based on 2 years of field experiments, we developed an interpretable artificial neural network model integrating 13 structural and physiological indicators to predict four quality parameters: vitamin C (VC), soluble sugar, titratable acid, and sugar‐acid ratio. The model architecture was optimized through Bayesian optimization, resulting in a 13–4–1/13–5–1 network structure with high prediction accuracy (*R* ^2^ = 0.89–0.98). Biological interpretation of the connection weights revealed that the elongation of bearing shoots (1.2–3.1 cm/month) and SPAD values (33–41.5) were key drivers of VC accumulation, reflecting their roles in photosynthate transport and light‐harvesting efficiency. Canopy structural characteristics, particularly leaf inclination angles of 26°–34° combined with a direct beam transmittance of 0.32–0.43, were found to synergistically enhance sugar accumulation by optimizing light distribution while maintaining sufficient gas exchange. Furthermore, net photosynthetic rates exceeding 12 μmol·m^−2^·s^−1^ significantly reduced organic acid content, indicating a shift in carbon partitioning toward sugar synthesis. These findings demonstrate that the model successfully bridges computational analysis with biological processes, providing both a predictive tool and mechanistic insights for gray jujube quality management. The integration of architectural, physiological, and environmental parameters in this framework offers a comprehensive approach for precision cultivation of this important crop.

## Graphical abstract

This study developed an interpretable neural network model to predict gray jujube quality (vitamin C, sugars, acids) using 13 tree‐structure and physiological indicators. Bayesian optimization achieved high accuracy (*R* ^2^ = 0.89–0.98), revealing key controls: pendant branch growth (1.2–3.1 cm/month) and SPAD (33–41.5) for VC, leaf angle (26°–34°) and light transmittance (0.32–0.43) for sugars, and photosynthesis (> 12 μmol m^−2^ s⁻¹) for acid reduction. The model enables precision cultivation of jujube orchards.

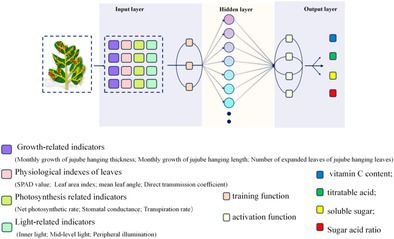

**Abbreviations**
ANN: Artificial Neural Network
MAE: Mean Absolute Error
MAPE: Mean Absolute Percentage Error
MBE: Mean Bias Error
MSE: Mean Squared Error
*R* ^2^
: Coefficient of Determination
RMSE: Root Mean Square Error
RPD: Relative Percent Difference

## 1 Introduction

Gray jujube ( *Ziziphus jujuba* Mill), as a characteristic economic forest fruit in the arid regions of Northwest China, has its market value and industrial benefits directly determined by fruit quality (Uddin et al. 2023). However, the formation of jujube flavor involves multidimensional interactions among tree architecture, physiological functions, and environmental factors, making it challenging for traditional statistical methods to decipher these complex nonlinear relationships (Wang et al. 2021). Particularly in the extreme arid environment of Xinjiang's Tarim Basin, jujube trees regulate fruit quality through unique morphological and physiological adaptation mechanisms, yet quantitative models of this process remain unexplored. Although machine learning technologies have demonstrated potential in crop phenotyping prediction, existing studies predominantly focus on single quality indicators, lacking systematic modeling of comprehensive flavor traits including vitamin C (VC), soluble sugars, organic acids, and sugar‐acid ratio. Moreover, the limited interpretability of these models severely restricts their agronomic guidance value.

The formation of jujube fruit flavor quality is comprehensively regulated by multiple growth and physiological indicators. Previous studies have shown that moderate increases in shoot diameter and length facilitate nutrient transport and photosynthetic area expansion, promoting the accumulation of soluble sugars and VC, while excessive growth may deplete carbon sources and reduce the sugar‐acid ratio (Liu et al. 2023). Higher SPAD values, net photosynthetic rates, and stomatal conductance significantly enhance photosynthetic efficiency and sugar synthesis (Xu et al. 2014), whereas leaf area index and mean leaf inclination angle indirectly regulate the sugar‐acid balance by modulating canopy light penetration (Liu et al. 2021). Reduced direct light transmittance and inner canopy illumination inhibit photosynthesis in shaded leaves, leading to decreased sugar content and increased organic acid accumulation, thereby lowering the sugar‐acid ratio (Tang et al. 2023). In contrast, strong peripheral light not only promotes sugar and VC synthesis but also accelerates acid degradation, significantly improving the sugar‐acid ratio and VC content in outer‐canopy fruits (Gautier et al. 2009). Moderate transpiration rates benefit nutrient transport and heat dissipation, whereas excessive rates may cause water stress and inhibit sugar accumulation (Chen et al. 2023). Collectively, optimizing canopy structure, balancing vegetative growth, and enhancing photosynthetic efficiency can effectively coordinate these synergistic interactions to ultimately improve jujube fruit flavor quality and nutritional value. Therefore, this study employs 13 indicators—monthly shoot diameter increment, shoot elongation, leaf expansion rate, SPAD value, leaf area index, mean leaf inclination angle, direct light transmittance coefficient, net photosynthetic rate, stomatal conductance, transpiration rate, and inner/mid/outer canopy irradiance—as predictive inputs for four flavor quality traits: soluble sugars, titratable acidity, VC content, and sugar‐acid ratio.

Given the nonlinear relationships between these 13 indicators and the four flavor quality traits, this study innovatively proposes an “Explainable ANN Framework” to deeply analyze these complex interactions. Previous research has demonstrated the limitations of traditional statistical methods in handling such relationships, while artificial neural networks (ANNs) have proven effective due to their powerful nonlinear modeling capabilities. In addressing nonlinear fitting challenges in apple growth prediction, Garriz et al. established a nonlinear logistic model based on longitudinal monitoring data (biweekly sampling of four fruits over five growing seasons) that accurately characterized three biological growth phases—initial cell division, rapid expansion (maximum growth rate 0.50 mm/day), and maturation plateau—achieving a superior fit (*R* ^2^ = 0.90, *p* < 0.001) compared to linear methods (Garriz et al. 2015). Similarly, Bargoti et al. validated the universal advantages of nonlinear algorithms in apple orchard yield prediction by integrating convolutional neural networks (CNNs) with watershed segmentation, achieving an F1‐score of 0.861 for single‐fruit detection and a yield prediction correlation of *R* ^2^ = 0.826 (Bargoti and Underwood 2016). Building on these advancements, this study innovatively combines Bayesian optimization with multiple training functions, intelligently optimizing network architecture and activation function combinations (logsig‐purelin) to significantly enhance prediction accuracy (*R* ^2^) while maintaining model interpretability. These innovations not only address the interpretability challenges of traditional ANN models in agricultural applications but also provide critical theoretical and practical guidance for precision agriculture optimization.

SHAP (Shapley Additive Explanations) analysis, a game theory‐based interpretability method for machine learning models, serves to quantify the contribution of each feature to model predictions, thereby elucidating the decision‐making mechanism. In practical applications, SHAP analysis achieves three primary objectives: (1) global interpretability—identifying key features and their directional effects; (2) local interpretability—explaining predictions for individual samples; and (3) feature interaction analysis—uncovering synergistic effects between features. In agricultural research, Shahood et al. employed random forest models coupled with SHAP interpretation to quantitatively analyze the dynamic relationship between sugar accumulation and malic acid degradation during grape berry maturation. Their findings revealed that the phloem sucrose unloading rate during the berry softening phase predominantly governs the sugar‐acid balance, with model predictions showing 87% concordance with single‐fruit physiological monitoring data. This study provided the first evidence at single‐fruit resolution that delayed initiation of sugar storage is a key source of fruit heterogeneity, demonstrating a stable 4:1 stoichiometric ratio between hexose accumulation and malic acid oxidation during the 2 weeks preceding maturity. The model further indicated that sugar concentration changes induced by water loss become the dominant quality factor during growth arrest (4 months post‐softening), offering theoretical guidance for precision harvesting (Shahood et al. 2020). In precision irrigation studies, Zhu et al. utilized principal component analysis to investigate the effects of different irrigation levels and drip depths on the tomato fruit sugar‐acid ratio (Soluble Sugar/Organic Acid Ratio). Their results demonstrated a remarkable correlation coefficient of 0.985 between sugar‐acid ratio and irrigation water use efficiency (IWUE), highlighting the decisive influence of water management on fruit flavor quality (Zhu et al. 2020). Furthermore, Duan et al. incorporated magneto‐electric water irrigation technology to quantify the interactive effects of water stress on apple fruit quality under extreme drought conditions. Their correlation matrix analysis revealed a negative association between the net photosynthetic rate (Pn) and fruit firmness (*r* = −0.38), while stomatal conductance (Gs) showed positive correlation with soluble solids content (*r* = 0.42), with model explanatory power reaching 91% (Duan et al. 2024). These findings collectively demonstrate that SHAP analysis not only enhances the transparency of agricultural models but also uncovers complex feature relationships that conventional statistical methods often fail to capture, thereby providing novel perspectives for agronomic optimization. In this study, we innovatively apply SHAP analysis to Chinese jujube quality modeling, aiming to overcome the “black box” limitations inherent in traditional machine learning approaches.

Concurrently, this study innovatively employs Response Surface Methodology (RSM) as a pivotal analytical approach, selected for its distinctive advantages in addressing complex agronomic system optimization challenges. Previous research has substantiated RSM's capability to overcome the limitations of conventional single‐factor experiments through constructing quantitative models of multifactorial interactions.

Ma et al. addressed the quantification challenge of water‐fertilizer coupling effects in wolfberry ( *Lycium barbarum* L.) orchards by developing a quadratic regression model (*R* ^2^ = 0.98) using Box–Behnken design, incorporating irrigation volume (X_1_) and nitrogen application rate (X_2_) as independent variables against yield (Y) (Ma et al. 2023; Meetiyagoda et al. 2023). Their analysis not only revealed that the X_1_X_2_ interaction term contributed 25% to yield variation (quantifying water‐nitrogen synergy through response surface analysis), but also identified optimal water‐nitrogen ratios (irrigation: 2500–2600 m^3^ ha^−1^; nitrogen: 220–230 kg ha^−1^). These optimal ranges, determined via comprehensive scoring methodology, maximized water use efficiency under Ningxia's arid‐semiarid conditions (Ma et al. 2023). Field implementation demonstrated a 7.48% yield increase compared to controls, alongside 3.73% and 5.2% improvements in dried fruit percentage and hundred‐grain weight, respectively, significantly enhancing both fruit quality and economic returns (Ma et al. 2023). In postharvest grape storage research, central composite design elucidated the effects of temperature (X_1_), relative humidity (X_2_), and O_2_ concentration (X_3_) on fruit quality. The established quadratic polynomial model (*R* ^2^ = 0.92) demonstrated that parameter combinations of (−1°C ± 0.3°C, 90% ± 2% RH, 5% ± 0.3% O_2_) extended storage duration to 60 days while maintaining decay rate below 4.8%, representing a 40% improvement over conventional refrigeration (He et al. 2009; Ke et al. 1991). These optimized conditions significantly preserved fruit quality by suppressing respiration rate (32% reduction) and Botrytis cinerea activity, maintaining fruit firmness (< 15% loss) and total soluble solids content (< 0.5% TSS decline), while retarding VC degradation (> 85% retention) (He et al. 2009).

These groundbreaking findings demonstrate that RSM can precisely capture nonlinear response relationships and parameter threshold effects in agricultural systems. Building upon this foundation, this study employs response surface analysis to establish reliable quadratic response surface models for the top four indicators identified through SHAP analysis. This approach will determine optimal parameter combination ranges, thereby providing quantifiable agronomic operation standards for (e.g., precision cultivation of gray jujube). The application of this methodology is expected to address key technical bottlenecks in current jujube production, particularly the lack of quantitative benchmarks for quality regulation.

To address these challenges, we propose an innovative framework integrating three methodological approaches: (1) Bayesian Optimization‐based adaptive selection of hidden layer nodes, ensuring model complexity aligns with physiological process hierarchies; (2) SHAP (SHapley Additive exPlanations) value analysis to elucidate feature contribution pathways and reveal nonlinear effects of key factors such as SPAD values and canopy light transmittance; (3) Construction of response surface models to quantify interaction thresholds among indicators. Through two‐year field trials (*n* = 144), we will measure 13 tree architecture indicators and 4 quality traits to address three core research questions: (1) How to construct ANN architectures adapted to the physiological characteristics of Chinese jujube; (2) Which tree architecture indicators predominantly drive quality variation; (3) Whether the models can provide quantitative decision‐making support for agronomic practices such as shoot thinning and foliar fertilization. This comprehensive research framework will provide novel methodological support for deciphering the mechanisms underlying gray jujube quality formation and developing precision cultivation technologies.

## 2 Materials and Methods

### 2.1 Experimental Design

The study was conducted at a jujube plantation in Xinjiang Production and Construction Corps (81°10′23′′E, 40°57′36′′N, elevation 1011 m), characterized by a typical continental arid desert climate. Key climatic parameters included: mean annual precipitation of 49 mm, annual sunshine duration of 2556.3 to 2991.8 h, mean annual temperature of 10.7°C, and frost‐free period of 207–220 days. The region exhibits distinctive climatic features of scarce precipitation, intense evaporation, significant diurnal temperature variation, and abundant solar radiation. The experimental site comprised flat terrain with deep, sandy loam soils, using traditional flood irrigation methods. The test materials were trunk‐shaped gray jujube ( *Ziziphus jujuba* Mill.) grafted onto wild jujube (Ziziphus spinosa Hu) rootstocks, planted in a north–south orientation with a spacing of 1.5 m (within rows) × 3 m (between rows).

### 2.2 Measurement of Growth Indicators of Jujube Pendant Branch

Six‐year‐old gray jujube trees were randomly selected from the orchard (*n* = 72). Five newly emerged pendant branches from the middle section of biennial secondary branches on each tree were tagged for measurement. From early June to early September in both 2021 and 2022, the pendant branches were measured at 15‐day intervals (six measurement sessions total). Three parameters were measured following standard methods (Xia et al. 2015), with three replicates per measurement point that were then averaged: (1) Branch diameter: Measured at 1 cm from the base using a digital caliper (Model CD‐15DCX, Mitutoyo Corporation, Japan; measuring range: 0–150 mm, resolution: 0.01 mm, accuracy: ±0.02 mm). (2) Branch length: Measured with a measuring tape (Model 34–272, Stanley Tools, USA; measurement range: 0–5 m, resolution: 1 mm, accuracy: ±1.3 mm at 20°C). (3) Leaf number: Determined by manual counting. All measurements were conducted by the same trained technician. After removing outliers using the interquartile range method, the means of six measurement periods were calculated (*n* = 360 per measurement session, comprising 72 trees × 5 branches).

### 2.3 Measurement of Canopy Architecture and Light Intensity

Canopy structural parameters were measured using the CI‐110 Plant Canopy Imager (CID Bio‐Science Inc., Camas, WA, USA) following standardized environmental conditions: measurements were conducted during stable weather periods (no precipitation for two consecutive days, wind speed < 2 m/s) under photosynthetically active radiation (PAR) levels of 800–1200 μmol·m^−2^·s^−1^ (calibrated with an LI‐190R quantum sensor) between 10:00 and 14:00 local time. Data collection was performed in mid‐July 2021 and 2022 under partly cloudy conditions (no direct intense sunlight or distinct shadows), with PAR fluctuations maintained within ±15% during measurement periods. Measurements were taken at four cardinal directions (east, south, west, and north) positioned 50 cm from the main trunk, using a fisheye lens with predetermined zenith angles (8.5°, 25.5°, 42.5°, 59.5°, and 76.5°) and azimuthal angles (0°–90°, 90°–180°, 180°–270°, and 270°–360°) for canopy image acquisition. The accompanying CI‐110 Software (v2.1) was used to calculate direct light transmittance, leaf area index (LAI), and mean leaf angle (MLA) (Chen et al. 2021). Each direction was measured in triplicate, with measurements repeated when the coefficient of variation exceeded 10%. Light intensity measurements were performed synchronously with canopy structure assessments using a certified TES‐1332A light meter (TES Electrical Electronic Corp., Taiwan) cross‐validated with an LI‐190R quantum sensor. Measurements were conducted at nine canopy positions per tree (inner, middle, and outer canopy at upper, middle, and lower layers), with the sensor maintained horizontally and parallel to the leaf surface. Three consecutive readings were averaged for each position, and all light intensity data were converted to PAR values (μmol·m^−2^·s^−1^) for statistical analysis.

### 2.4 Determination of Leaf Photosynthetic Parameters and SPAD Values

The photosynthetic parameters were measured on clear days (August 15, 2021 and August 17, 2022) using mature functional leaves collected from the middle section of current‐year pendant branches in the south‐facing canopy. Measurements were performed with a LI‐6400 portable photosynthesis system (LI‐COR Biosciences, Lincoln, NE, USA) to determine: net photosynthetic rate (Pn, μmol m^−2^ s^−1^), stomatal conductance (Gs, mmol m^−2^ s^−1^), intercellular CO_2_ concentration (Ci, μmol mol^−1^), and transpiration rate (Tr, mmol m^−2^ s^−1^). Three stable readings were recorded per tree with three biological replicates. The water use efficiency (WUE) was calculated based on the recorded parameters, using the following formula:

WUE=Pn÷Tr

Simultaneously, leaf chlorophyll content was assessed using a SPAD‐502 chlorophyll meter (Konica Minolta, Japan) by measuring 10 functional leaves from each cardinal direction (*n* = 40 leaves per tree). All measurements were conducted by the same technician between 08:00 and 11:00 local time under stable environmental conditions to minimize diurnal variations.

### 2.5 Determination of Internal Quality of Fruits

Determination of soluble sugar: The anthrone colorimetric method is used (Wu and Shen 2021). Take 0.5 g of fresh sample, grind it with liquid nitrogen, add 10 mL of extraction solution, and conduct 30 min of water bath extraction at 80°C. After cooling, centrifuge (10,000 ***g*** for 10 min), and combine the supernatant to make up to 25 mL. During color development, take 0.2 mL of the extraction solution, add 5 mL of freshly prepared 0.2% anthrone‐sulfuric acid reagent, and heat precisely in a boiling water bath for 10 min, then cool in an ice bath. Use the blank reagent as the reference, measure the absorbance (at 620 nm) with a spectrophotometer, and calculate the content based on the glucose standard curve (0–100 μg/mL, *R* ^2^ ≥ 0.999). The calculation formula is:

$$\text{Soluble sugar content}\;\left(\mathrm{mg}/g\right)=\left(C\times V\right)/\left(W\times 10^{3}\right)$$

Here, C represents the standard curve concentration (in μg/mL), V represents the volume of the extract (in mL), and W represents the fresh weight of the sample (in g).

Determination of titratable acid: The method using sodium hydroxide solution titration was employed (S. Zhu et al. 2023). Take 5.0 g of homogenized sample, add 50 mL of de‐CO₂ water, conduct boiling water bath extraction for 30 min, make up to 100 mL, and then centrifuge (10,000 ***g*** for 15 min). Filter the supernatant through a 0.45 μm filter membrane. Take 10 mL of the filtrate, add 2 drops of phenolphthalein, titrate with NaOH until a faint red color appears and does not fade within 30 s. Record the consumed volume (V_1_), and the consumed volume of the blank control is V_0_. The content of titratable acid is calculated as:

$$\text{Content}\;\left(g/\mathrm{kg}\right)=\left(V_{1}-V_{0}\right)\times \text{CNaOH}\times 0.064\times 10/\text{Sample fresh weight}\;\left(\mathrm{kg}\right)$$

V_1_ represents the volume of NaOH consumed in the sample titration (in liters), while V_0_ represents the volume of NaOH consumed in the blank test (also in liters). The difference between these two values is used to eliminate background interference. CNaOH is the concentration of the NaOH standard solution (in moles per liter).

Determination of Vitamin C: The method using molybdenum blue colorimetry was employed (Liu et al. 2019). 1.0 g of fresh sample was ground with liquid nitrogen, then 10 mL of pre‐cooled extraction solution was added and homogenized in an ice bath. The mixture was centrifuged at 4°C for 15 min at 12,000 ***g***. The supernatant was filtered through a 0.22 μm filter membrane. For color development, 1.0 mL of the filtrate was taken, and 1.0 mL of 5% ammonium molybdate solution and 2.0 mL of 0.15 M sulfuric acid were added. The mixture was heated in a boiling water bath for 10 min precisely and then cooled in an ice bath. The volume was adjusted to 5 mL. The absorbance was measured using the blank reagent (1 mL of the extraction solution instead of the sample) as the reference. The content was calculated based on the ascorbic acid standard curve (0–100 μg/mL, *R* ^2^ ≥ 0.998). The formula is:

$$\text{Vitamin}\;C\;\text{content}\;\left(\mathrm{mg}/g\right)=C\times V/W\times 10^{3}$$

where C represents the standard curve concentration (μg/mL), V represents the volume of the extraction solution (mL), W represents the fresh weight of the sample (g), 10^3^ is the conversion factor from μg to mg.

Calculation of sugar‐to‐acid ratio (Li et al. 2021):

Sugar−to−acid ratio=Soluble sugar/Titratable acid

### 2.6 Statistical Analysis

In this study, data modeling was conducted using MATLAB software (version R 2024b, MathWorks Inc., Natick, MA, USA).

#### 2.6.1 Construction of Artificial Neural Network

**FIGURE 1:**
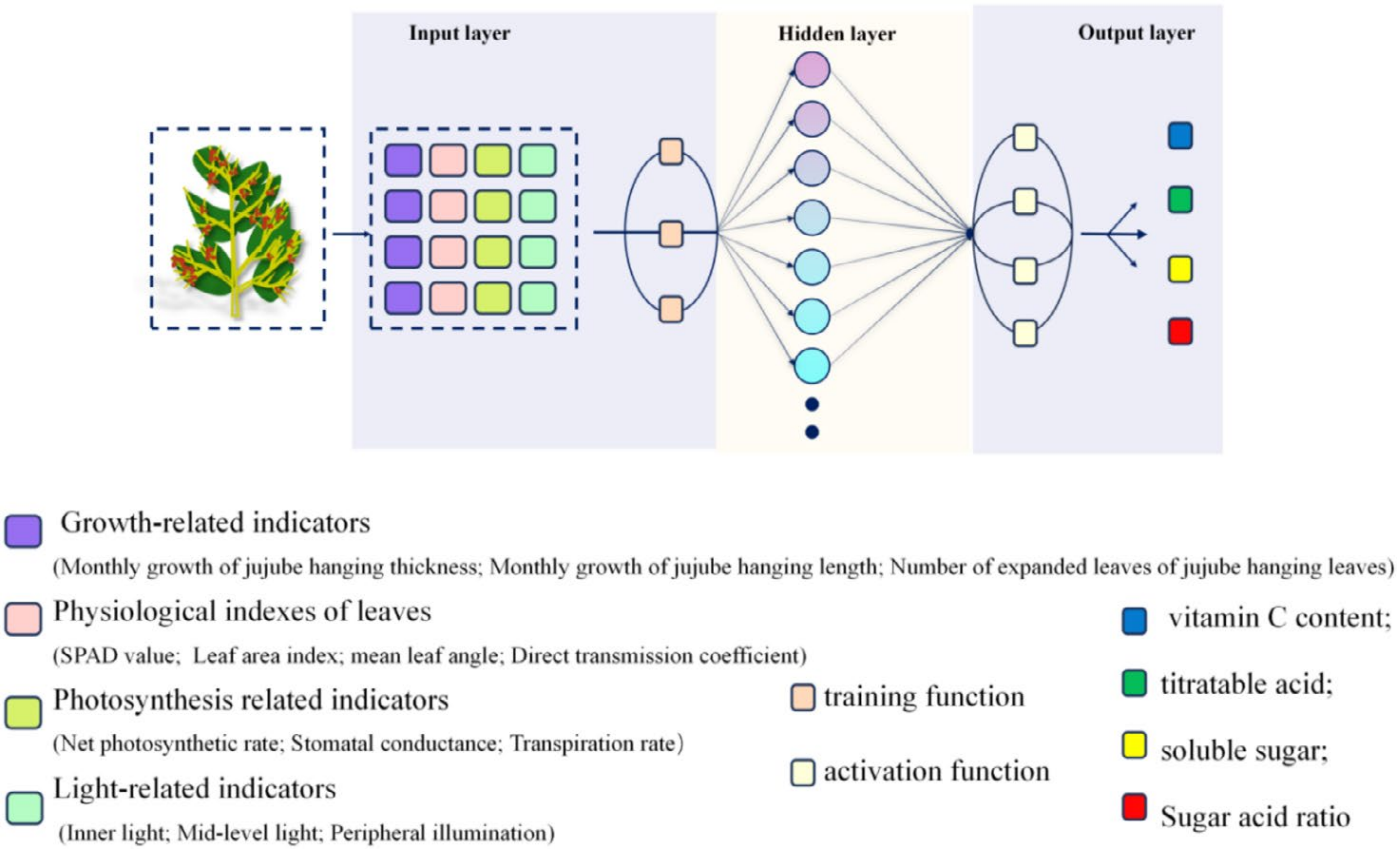
Experimental flowchart.

In this study, 13 tree structure and physiological function indicators, including the monthly increase in the thickness of the jujube branches, the growth of the branch length, the number of leaf expansions on the branches, the SPAD value, the leaf area index, the average leaf inclination angle, the direct transmittance coefficient, the net photosynthetic rate, the stomatal conductance, the transpiration rate, the inner light intensity, the middle layer light intensity, and the peripheral light intensity, were used as inputs, and the four fruit quality indicators of VC, soluble sugar, titratable acid, and sugar–acid ratio of the gray jujube fruits were used as outputs (Figure 1). Three training functions (trainlm, trainbr and traingdx), five hidden layer activation functions (tansig, poslin, logsig, purelin and radbas) and four output layer activation functions (purelin, poslin, logsig and tansig). To elucidate the model's decision‐making mechanism, SHAP (SHapley Additive exPlanations) analysis was performed using the Explainable Neural Network Regression Model with SHAP plugin (v2.1.0) in MATLAB R2024b. The key parameter configurations were as follows: background samples were selected through stratified random sampling (80 representative samples, accounting for 55.6% of the total training set); the kernel function utilized the default shapleyKernel (weighted linear LIME kernel) with bandwidth parameters optimized via Silverman's rule; and the feature perturbation mode employed the “Interventional” approach (based on marginal distribution sampling) with a maximum permutation number set at 5 × 10^4^.

Trainlm (Levenberg–Marquardt Backpropagation) Weight Update Rule Formula:

(1) $$∆\omega ={\left(J^{T}J+\mathrm{\mu I}\right)}^{-1}J^{T}e$$

J is the Jacobian matrix of the error with respect to the weights; *μ* is the damping factor (adjustable to control the balance between gradient descent and Newton's method); e is the error vector; I is the identity matrix.

Trainbr (Bayesian regularization algorithm) The objective function for the improvement of the framework:

(2) $$F\left(w\right)=\beta E\_D+\alpha E\_w$$

E_D is the sum of squared errors; E_w is the sum of squared weights; *α* and *β* are hyperparameters (Automatically optimize through the evidence framework).

Traingdx (Adaptive learning rate gradient descent with variable quantities) The weight.

Update rule includes momentum terms and adaptive learning rate.

(3) $$v\left(t\right)=\mathrm{\gamma v}\left(t-1\right)-\eta \left(t\right)\nabla E\left(w\left(t\right)\right)$$

(4) $$w\left(t+1\right)=w\left(t\right)+v\left(t\right)$$

*v*(*t*) represents the weight update momentum at the current moment; γ is the momentum coefficient (default 0.9); *η*(*t*) is the adaptive learning rate; ∇E is the gradient; *w*(*t*) is the weight parameter value of the current iteration step.

Tansig (Hyperbolic tangent S‐type):

(5) $$f\left(x\right)=\tanh\left(x\right)=\frac{e^{x}-e^{-x}}{e^{x}+e^{-x}}$$

Purelin (Linear):

(6) $$f\left(x\right)=x$$

Logsig (Logarithmic S‐shaped):

(7) $$f\left(x\right)=\frac{1}{1+e^{-x}}$$

Purelin (Positive Linearity):

(8) $$\text{poslin}\left(x\right)=\max\left(0,x\right)=\left\{\begin{array}{c} x\;\text{if}\;x\geq 0\\ 0\;\text{if}\;x\leq 0 \end{array}\right.$$

Radbas (Radial basis):

(9) $$\text{radbas}\left(x\right)=e^{-x^{2}}$$

#### 2.6.2 Artificial Neural Network Optimization

**FIGURE 2:**
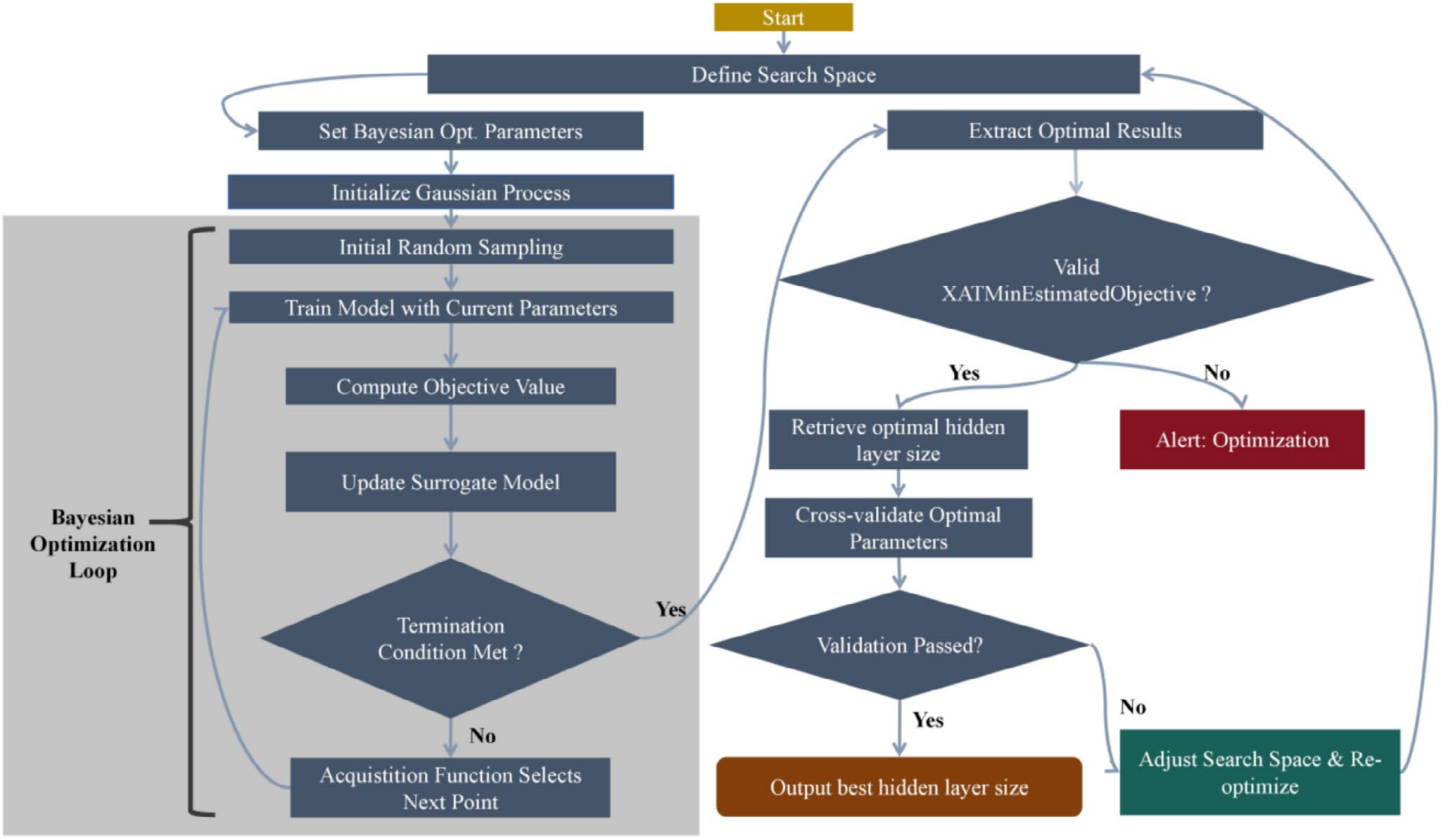
Flowchart of the Bayesian optimization algorithm for determining the optimal number of hidden layer nodes in the artificial neural network.

The optimal number of hidden layer nodes in the artificial neural network is determined using the Bayesian optimization algorithm. Firstly, the target function CostFunction is defined to evaluate the network performance, and the optimization range of the number of hidden layer nodes is set to be integer values from 4 to 20. The optimization process is executed using the bayesopt function, with the following parameter settings: no limit on the maximum optimization time (MaxTime = Inf), 50 maximum evaluation times (MaxObjectiveEvaluations = 50), the target function allows for randomness (IsObjectiveDeterministic = false), display detailed optimization information (Verbose = 1), and disable parallel computing to ensure the reproducibility of the results (UseParallel = false). After the optimization is completed, the optimal number of hidden layer nodes that minimizes the target function (Best hidden layer size) is extracted from the BayesObject results. This method achieves efficient parameter optimization by constructing a Gaussian process surrogate model and using the acquisition function to guide the search (Figure 2). During optimization, we employed multiple random initializations (seed values: 42, 123, 456, 789, 2023, 1001, 314,159, 2718, 1618, 777) to examine solution robustness. The final selected network architecture was then applied to fruit quality prediction modeling.

We tested different training functions and transfer functions to find the final model, and determined the coefficients (*R* ^2^), root mean square error (RMSE), mean bias error (MBE), mean absolute error (MAE), mean absolute percentage error (MAPE), mean squared error (MSE), and relative analysis error (RPD). The equations are as follows:

(10) $$R^{2}=\frac{\sum_{i=1}^{n}\left(M_{i}-\bar{M}\right)\left(P_{i}-\bar{P}\right)}{\sqrt{\sum_{i=1}^{n}{\left(M_{i}-\bar{M}\right)}^{2}\sum_{i=1}^{n}{\left(P_{i}-\bar{P}\right)}^{2}}}$$

(11) $$\mathrm{MAE}=\frac{1}{n}\sum_{i=1}^{n}\left|M_{i}-P_{i}\right|$$

(12) $$\mathrm{MBE}=\frac{1}{n}\sum_{i=1}^{n}\left(M_{i}-P_{i}\right)$$

(13) $$\text{MAPE}=\frac{100\%}{n}\sum_{i=1}^{n}\left|\frac{M_{i}-P_{i}}{M_{i}}\right|$$

(14) $$\text{RMSE}=\sqrt{\frac{1}{n}\sum_{i=1}^{n}{\left(M_{i}-P_{i}\right)}^{2}}$$

(15) $$\mathrm{MSE}=\frac{1}{n}\sum_{i=1}^{n}{\left(M_{i}-P_{i}\right)}^{2}$$

(16) $$\mathrm{RPD}=\frac{\mathrm{Std}\left(M_{i}\right)}{\text{RMSE}}$$

M_*i*_ is the actual observed value of the *i* sample in the dataset; P_*i*_ is the predicted output value of the *i* sample in the dataset.

## 3 Results and Analysis

### 3.1 Visual Analysis of Input Indicators and Output Indicators

**FIGURE 3:**
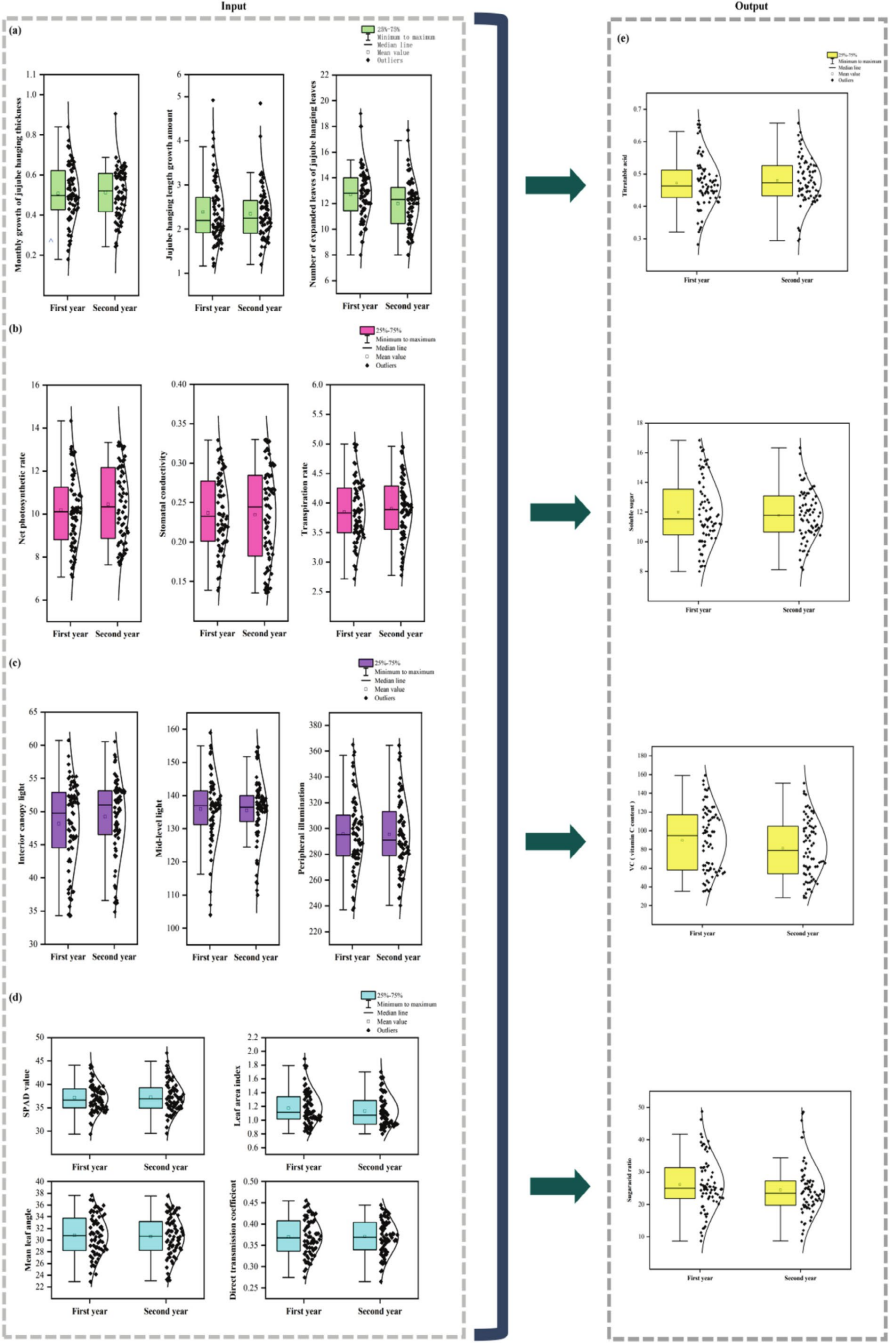
Visualization of input indicators and output indicators.

This study developed prediction models for jujube fruit quality (ascorbic acid, soluble solids, titratable acidity, and sugar‐to‐acid ratio) using 13 structural and physiological indicators across four categories: growth morphology (Figure 3a), leaf functionality (3b), canopy architecture (3c), and light environment (3d), with quality parameters shown in Figure 3e. Normality tests confirmed all parameters followed normal distributions. Interannual analysis revealed consistent patterns between study years without significant variations, while substantial intra‐annual variability provided diverse feature dimensions that enhanced model generalization and robustness (Figure 3).

### 3.2 Gray Jujube VC Model Based on ANN Algorithm

**FIGURE 4:**
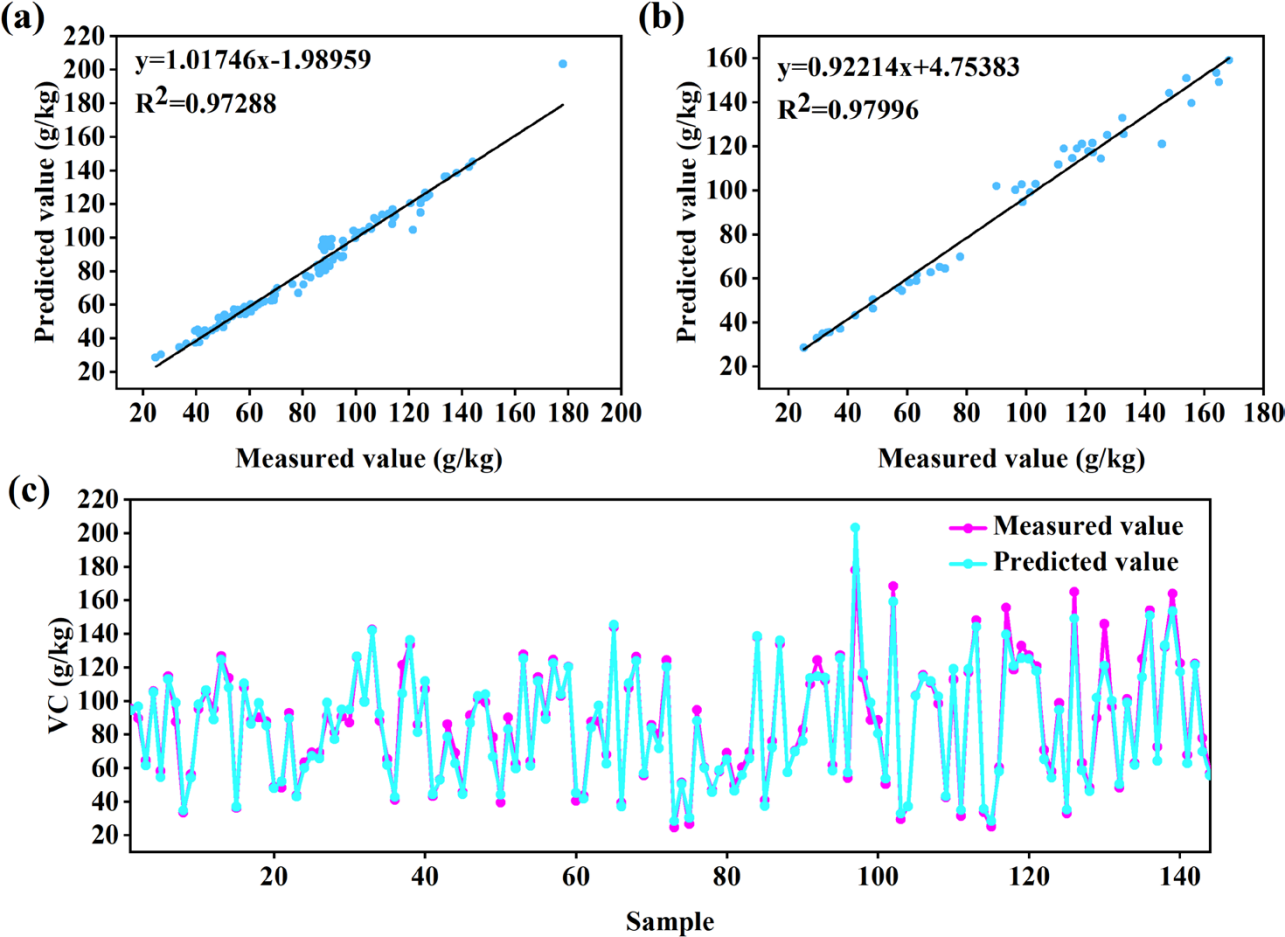
Results of the gray jujube VC model based on ANN algorithm: (a) Linear fitting graph of the training set; (b) Linear fitting of the validation set; (c) Line graph of the measured and predicted fruit indicators during the testing phase of the artificial neural network (The *X*‐axis shows the scale (0–140); the actual sample size = 144).

Comparative analysis of three neural network models (Table 1) revealed that the logsig‐Purelin dual‐hidden‐layer network constructed with the Levenberg–Marquardt algorithm (trainlm) demonstrated superior predictive performance. The model exhibited robust prediction accuracy in both training and validation sets (*R* ^2^ ≈ 0.97), with significantly better validation metrics including root mean square error (RMSE ≈ 6.9526), relative percent difference (RPD ≈ 6.0827), mean absolute error (MAE ≈ 4.9399), and mean absolute percentage error (MAPE ≈ 5.62%), while maintaining acceptable systematic bias (MBE ≈ 2.6131), confirming excellent generalization capability. While the Bayesian regularization algorithm (trainbr) achieved higher training set accuracy (*R* ^2^ ≈ 0.98), its validation performance declined (RMSE ≈ 8.00), indicating potential overfitting. The traditional gradient descent algorithm (traingdx) showed greater systematic deviation in validation (MBE ≈ 3.5117), suggesting inferior prediction stability. Validation confirmed the optimal 13–4–1 architecture (Figure 10a), with strong agreement between predicted and measured VC content (training *R* ^2^ ≈ 0.97288, validation *R* ^2^ ≈ 0.97996; Figure 4a,b). The close distribution matching (Figure 4c) demonstrates the model's effectiveness in integrating jujube tree structural and physiological indicators for accurate VC content prediction. All predictive models for vitamin C (VC) content developed in this study are provided in Supporting Information S1, Table 1.

**TABLE 1:** Gray dates VC model indicators based on ANN algorithm.

Training function	Training function	Connection function	*R* ^2^	RMSE	MSE	RPD	MAE	MBE	MAPE
Traingdx	Purelin‐Purelin	Training set	0.96	6.5559	42.9792	4.8231	5.2365	0.5558	0.0820
		Validation set	0.95	8.8439	78.2142	4.7747	6.0604	3.5117	0.0825
Trainbr	Poslin‐Purelin	Training set	0.98	4.7596	22.6533	7.0298	2.7480	0.0554	0.0380
		Validation set	0.95	8.0005	64.0073	4.5593	5.4689	1.3160	0.0920
Trainlm	Logsig‐Purelin	Training set	0.97	5.2883	27.9660	6.0407	3.8511	0.5251	0.0492
		Validation set	0.97	6.9526	48.3390	6.0827	4.9399	2.6131	0.0562

Abbreviations: MAE, Mean absolute error; MAPE, Mean absolute percentage error; MBE, Mean bias error; MSE, Mean squared error; *R* ^2^, Coefficient of determination; RMSE, Root mean square error; RPD, Relative percent difference.

### 3.3 Model of Soluble Sugars in Gray Jujube Based on ANN Algorithm

**FIGURE 5:**
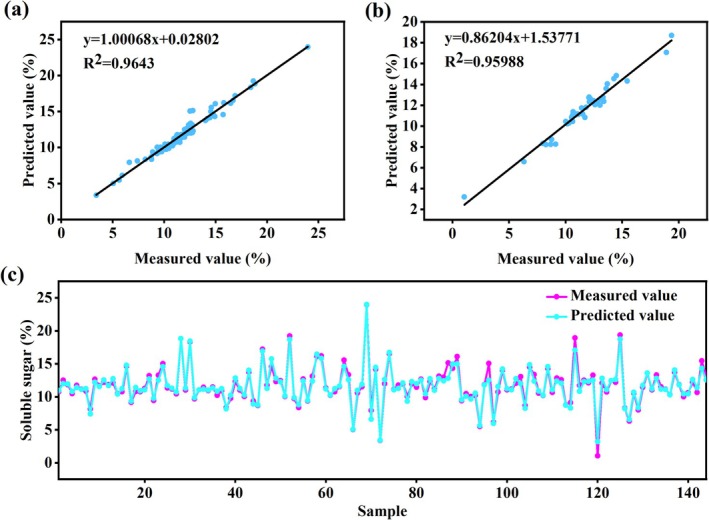
Results of the soluble sugar model of gray jujube based on ANN algorithm: (a) Linear fitting graph of the training set; (b) Linear fitting of the validation set; (c) Line graph of the measured and predicted fruit indicators during the testing stage of the artificial neural network (The *X*‐axis shows the scale (0–140); the actual sample size = 144).

The evaluation results (Table 2) demonstrated excellent model performance, with high fitting accuracy in the training set (*R* ^2^ ≈ 0.98, RMSE ≈ 0.4698) and superior predictive capability (RPD ≈ 6.3593). Although moderate performance decline was observed in validation (*R* ^2^ ≈ 0.93, RMSE ≈ 0.6685, RPD ≈ 3.8761), all parameters remained within acceptable ranges, confirming good generalization ability. Notably, the minimal mean bias error (MBE ≈ 0.0144) in validation underscored the model's prediction stability. Comparative analysis revealed distinct advantages of the Bayesian regularization approach over conventional methods: the gradient descent algorithm (traingdx) exhibited conservative predictions (*R* ^2^ ≈ 0.89), while the Levenberg–Marquardt algorithm (trainlm) showed greater systematic deviation (MBE ≈ 0.08). The optimal 13–4–1 architecture was determined through model computation (Figure 10b). Validation results demonstrated strong agreement between predicted and measured values, with *R* ^2^ values of 0.9643 (training) and 0.95988 (validation) (Figure 5a,b). The close distribution matching (Figure 5c) confirmed the model's effectiveness in integrating tree structural and physiological indicators for accurate soluble sugar content prediction. All predictive models for soluble solids content (SSC) developed in this study are provided in Supporting Information S1, Table 2.

**TABLE 2:** Model indicators of soluble sugar in jujube based on ANN algorithm.

Training function	Training function	Connection function	*R* ^2^	RMSE	MSE	RPD	MAE	MBE	MAPE
Traingdx	Radbas‐purelin	Training set	0.88	0.9919	0.9839	2.9111	0.7430	0.0178	0.0632
		Validation set	0.89	0.9528	0.9078	3.0805	0.6955	0.0350	0.0609
Trainbr	Poslin‐Purelin	Training set	0.98	0.4698	0.2207	6.3593	0.3421	0.0118	0.0281
		Validation set	0.93	0.6685	0.4469	3.8761	0.4816	0.0144	0.0748
Trainlm	Logsig‐Purelin	Training set	0.95	0.6249	0.3905	4.6760	0.4096	0.0825	0.0368
		Validation set	0.93	0.7649	0.5850	3.8277	0.5909	0.0861	0.0474

Abbreviations: MAE, Mean absolute error; MAPE, Mean absolute percentage error; MBE, Mean bias error; MSE, Mean squared error; *R* ^2^, Coefficient of determination; RMSE, Root mean square error; RPD, Relative percent difference.

### 3.4 Gray Jujube Titratable Acid Model Based on ANN Algorithm

**FIGURE 6:**
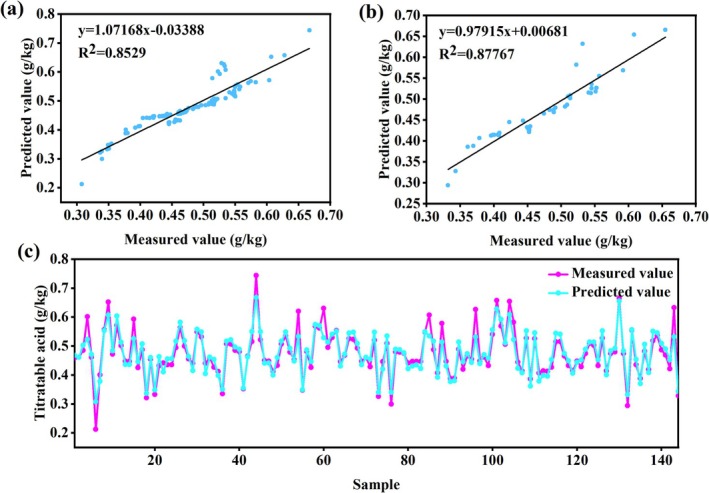
Results of the ANN algorithm‐based model for the titratable acid of gray jujube (a): Linear fitting graph of the modeling set; (b): Linear fitting of the validation set; (c): Line graph of the measured and predicted fruit indicators in the artificial neural network testing stage (The *X*‐axis shows the scale (0–140); actual sample size = 144).

Comparative analysis of model performance (Table 3) demonstrated that the radial basis‐tansig network constructed using Bayesian regularization (trainbr) exhibited optimal predictive capability. The model maintained consistent performance in both training (*R* ^2^ ≈ 0.89, RMSE ≈ 0.0280) and validation sets (*R* ^2^ ≈ 0.87, RMSE ≈ 0.0272), with relative percent difference (RPD > 2.8306) confirming its robust generalization ability. The Levenberg–Marquardt algorithm (trainlm) achieved the highest *R* ^2^ in validation (0.90), but the discrepancy between training and validation *R* ^2^ (*R* ^2^ ≈ 0.06) coupled with significant mean bias error (MBE ≈ 0.0031) suggested potential overfitting. The traditional gradient descent algorithm (traingdx) showed inferior performance (*R* ^2^ ≈ 0.70–0.74, RPD < 2). The optimal 13–4–1 architecture was determined through model computation (Figure 10c). Validation results demonstrated excellent agreement between predicted and measured values, with *R* ^2^ values of 0.8529 (training) and 0.87767 (validation) (Figure 6a,b). The close correspondence between predicted and actual measurements across all 144 samples (Figure 6c) confirmed the model's effectiveness in integrating tree architectural characteristics and physiological indicators for accurate titratable acidity prediction. Science and Technology Bureau of Xinjiang Production and Construction Corps. All predictive models for titratable acidity developed in this study are provided in Supporting Information S1, Table 3.

**TABLE 3:** Model indicators for titratable acid of Gray Jujube based on ANN algorithm.

Training function	Training function	Connection function	*R* ^2^	RMSE	MSE	RPD	MAE	MBE	MAPE
Traingdx	Purelin‐tansig	Training set	0.74	0.0365	0.0013	1.9620	0.0283	0.0005	0.0587
		Validation set	0.70	0.0509	0.0026	1.8153	0.0364	0.0014	0.0833
Trainbr	Radbas‐tansig	Training set	0.89	0.0280	0.0008	2.8562	0.0208	0.0007	0.0431
		Validation set	0.87	0.0272	0.0007	2.8306	0.0203	0.0071	0.0418
Trainlm	Purelin‐Purelin	Training set	0.84	0.0298	0.0009	2.5394	0.0194	0.0023	0.0426
		Validation set	0.90	0.0272	0.0007	3.1106	0.0194	0.0031	0.0411

Abbreviations: MAE, Mean absolute error; MAPE, Mean absolute percentage error; MBE, Mean bias error; MSE, Mean squared error; *R* ^2^, Coefficient of determination; RMSE, Root mean square error; RPD, Relative percent difference.

### 3.5 Gray Jujube Sugar‐Acid Ratio Model Based on ANN Algorithm

**FIGURE 7:**
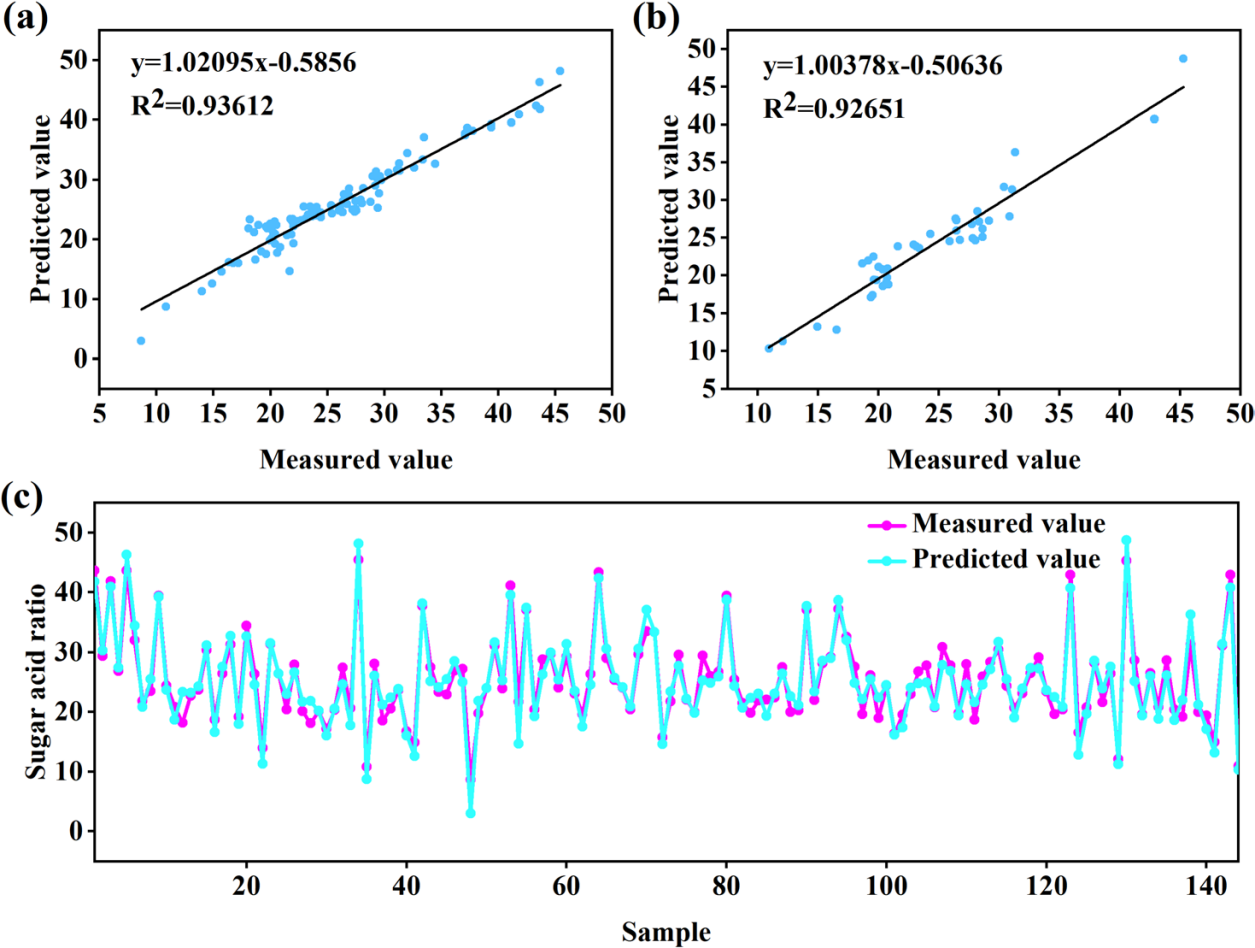
Results of the gray jujube sugar‐acid ratio model based on ANN algorithm: (a) Linear fitting graph of the training set; (b) linear fitting of the validation set; (c) line graph of the measured and predicted fruit indicators during the testing phase of the artificial neural network (The X‐axis shows the scale (0–140); the actual sample size = 144).

Comparative analysis (Table 4) demonstrated that the Purelin‐Purelin network constructed using Bayesian regularization (trainbr) achieved optimal predictive performance. The model exhibited excellent fitting capability in the training set (*R* ^2^ ≈ 0.95, RMSE ≈ 1.7329) while maintaining stable prediction accuracy in validation (*R* ^2^ ≈ 0.91, RMSE ≈ 1.9847). Notably, its relative percent difference values (RPD ≈ 4.6770 for training, RPD ≈ 3.3084 for validation) significantly exceeded the critical threshold of 3.0, confirming superior generalization ability. The Levenberg–Marquardt algorithm (trainlm) showed slightly higher validation *R* ^2^ (0.93), but the mean bias error difference between training and validation sets (MBE ≈ 0.1080) indicated systematic deviation potentially caused by overfitting. The traditional gradient descent algorithm (traingdx) performed inadequately (validation RPD ≈ 2.8640), failing to meet benchmark requirements. Validation confirmed the optimal 13–5–1 architecture (Figure 10d), with strong agreement between predicted and measured values (training *R* ^2^ ≈ 0.93612, validation *R* ^2^ ≈ 0.92651; Figure 7a,b). The high consistency between predicted and actual measurements across all 144 samples (Figure 7c) demonstrated the model's effectiveness in integrating tree structural and physiological indicators for accurate sugar‐acid ratio prediction. All predictive models for sugar‐acid ratio developed in this study are provided in Supporting Information S1, Table 4.

**TABLE 4:** Gray jujube sugar‐acid ratio model indicators based on ANN algorithm.

Training function	Training function	Connection function	*R* ^2^	RMSE	MSE	RPD	MAE	MBE	MAPE
Traingdx	Purelin‐Purelin	Training set	0.92	2.0857	4.3500	3.5832	1.6568	0.0083	0.0703
		Validation set	0.88	2.8283	7.9992	2.8640	2.2169	0.1083	0.0978
Trainbr	Purelin‐Purelin	Training set	0.95	1.7329	3.0030	4.6770	1.2820	0.0461	0.0599
		Validation set	0.91	1.9847	3.9391	3.3084	1.5192	0.1679	0.0629
Trainlm	Purelin‐tansig	Training set	0.93	1.8998	3.6092	3.8801	1.4886	0.3495	0.0734
		Validation set	0.93	2.3365	5.4593	3.7371	1.6023	0.4575	0.0756

Abbreviations: MAE, Mean absolute error; MAPE, Mean absolute percentage error; MBE, Mean bias error; MSE, Mean squared error; *R* ^2^, Coefficient of determination; RMSE, Root mean square error; RPD, Relative percent difference.

### 3.6 SHAP Interpretability Analysis

**FIGURE 8:**
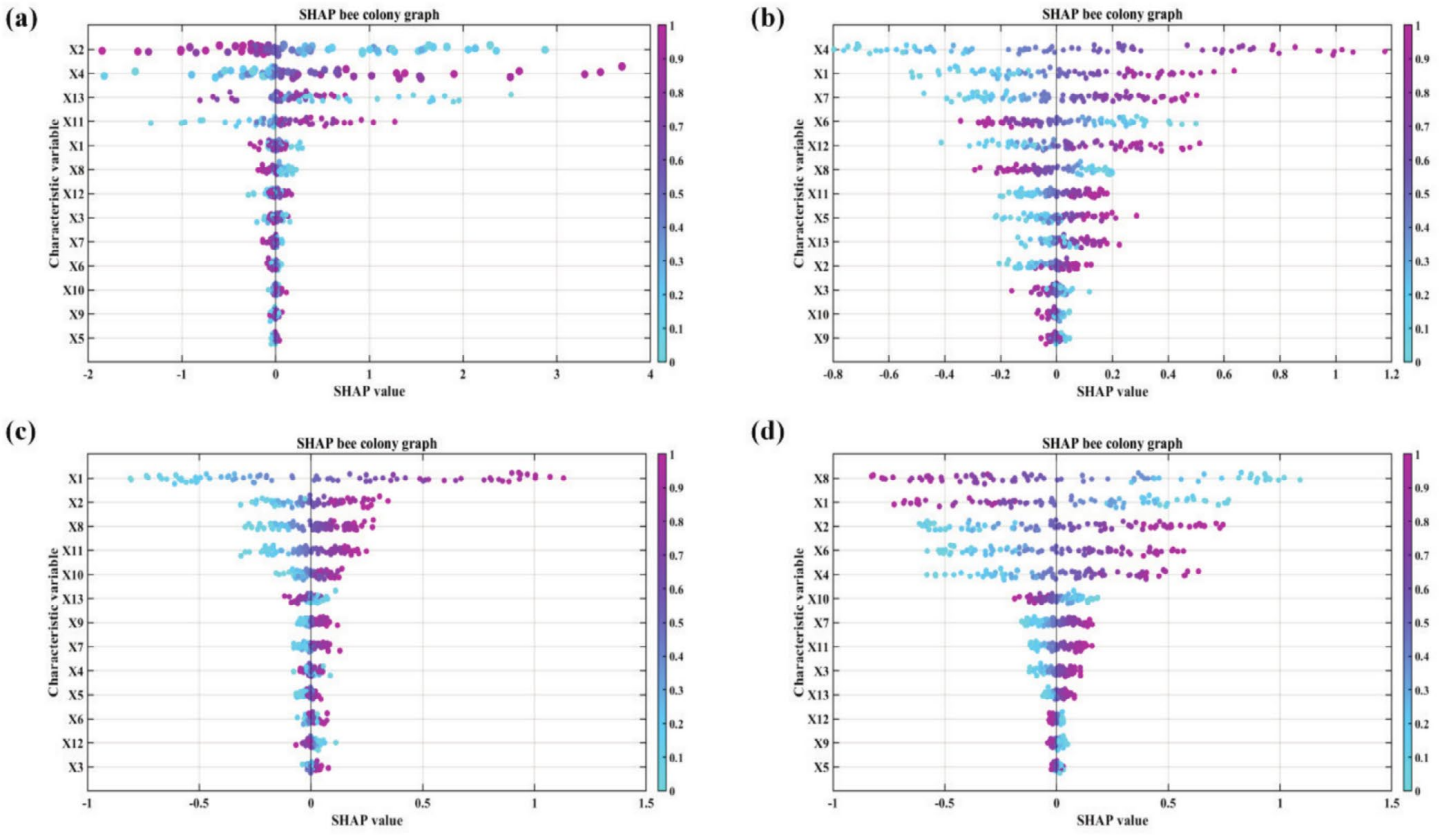
Interpretability analysis of SHAP based on ANN model (a) VC; (b) Soluble sugar; (c) Titratable acid; (d) Sugar–acid ratio.

A comprehensive analysis of the SHAP bee swarm plots revealed distinct feature contribution patterns across the four prediction models. In the VC prediction model (Figure 8a), X2 exhibited a broad SHAP value range of [−1.2, 1.8], demonstrating its capacity to either significantly increase or decrease predicted VC values depending on sample characteristics, reflecting a complex nonlinear influence mechanism. X4 showed similarly dynamic regulation with SHAP values distributed between [−0.8, 1.5], where the magnitude and direction of its contributions varied substantially across samples. In contrast, X13 consistently provided positive contributions SHAP: [0.2, 0.6] while X11 displayed context‐dependent effects SHAP: [−0.4, 0.5], alternating between enhancement and suppression based on sample properties.

The soluble sugar prediction model (Figure 8b) was most strongly influenced by X4, which showed the widest SHAP value range [−2.1, 2.3] and could dramatically elevate or reduce predictions at its extremes. X1 maintained stable bidirectional regulation SHAP: [−1.3, 1.6], whereas X7 [0.3, 0.9] and X6 [0.2, 0.8] primarily contributed positively but exhibited occasional contribution reversals in specific samples, highlighting their conditional effects.

**FIGURE 9:**
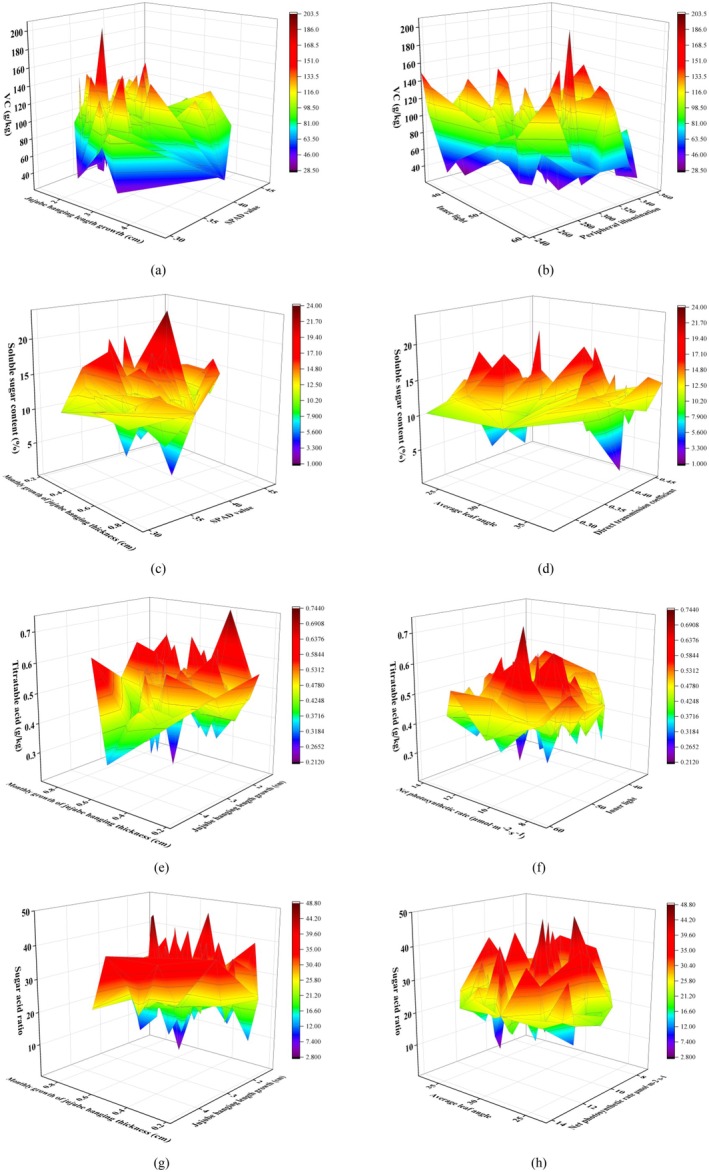
Response surface analysis: (a) The monthly growth of jujube hanging length, SPAD value, and VC content; (b) inner light intensity, peripheral light intensity, and VC content; (c) the monthly growth of jujube hanging diameter, SPAD value, and soluble sugar; (d) average leaf inclination angle, direct transmittance, and soluble sugar; (e) the monthly growth of jujube hanging diameter, the monthly growth of jujube hanging length, and titratable acid; (f) net photosynthetic rate, light intensity, and titratable acid; (g) the monthly growth of the thickness of the jujube hanging, the growth of the length of the jujube hanging, and the sugar acid ratio; (h) average leaf angle, net photosynthetic rate, and sugar‐acid ratio.

For the titratable acid model (Figure 9c), X1 emerged as the dominant feature with a distinct bimodal SHAP distribution [−1.5, 1.7], where both positive and negative extremes significantly impacted predictions. X2 showed similarly broad regulatory effects SHAP: [−1.1, 1.4]. While X8 [0.4, 1.0] and X11 [−0.3, 0.7] generally promoted predictions, they demonstrated complex sample‐specific behaviors, including occasional effect reversals.

In the sugar‐acid ratio model (Figure 8d), X8 served as the primary driver with the most extensive SHAP range [−2.5, 2.8], capable of substantially increasing or decreasing predictions. X1 maintained consistent bidirectional regulation SHAP: [−1.4, 1.8], while X6 [0.3, 0.9] and X4 [−0.6, 1.2] showed predominantly positive but variable contributions that could weaken or reverse under certain conditions.

### 3.7 Response Surface Analysis

The response surface analysis, based on the top four key features identified through SHAP analysis of the interpretable ANN model, systematically investigated the effects of jujube tree architecture and physiological indicators on fruit nutritional and flavor quality. For VC content optimization, optimal parameter ranges were identified as bearing shoot elongation (1.2–3.1 cm/month), SPAD value (33.35–41.5), inner canopy irradiance (34.45–57.75 μmol·m^−2^·s^−1^), and outer canopy irradiance (237–342.33 μmol·m^−2^·s^−1^), with simultaneous optimization of these parameters yielding peak VC content of 203.42 mg/kg (Figure 9a,b).

**FIGURE 10:**
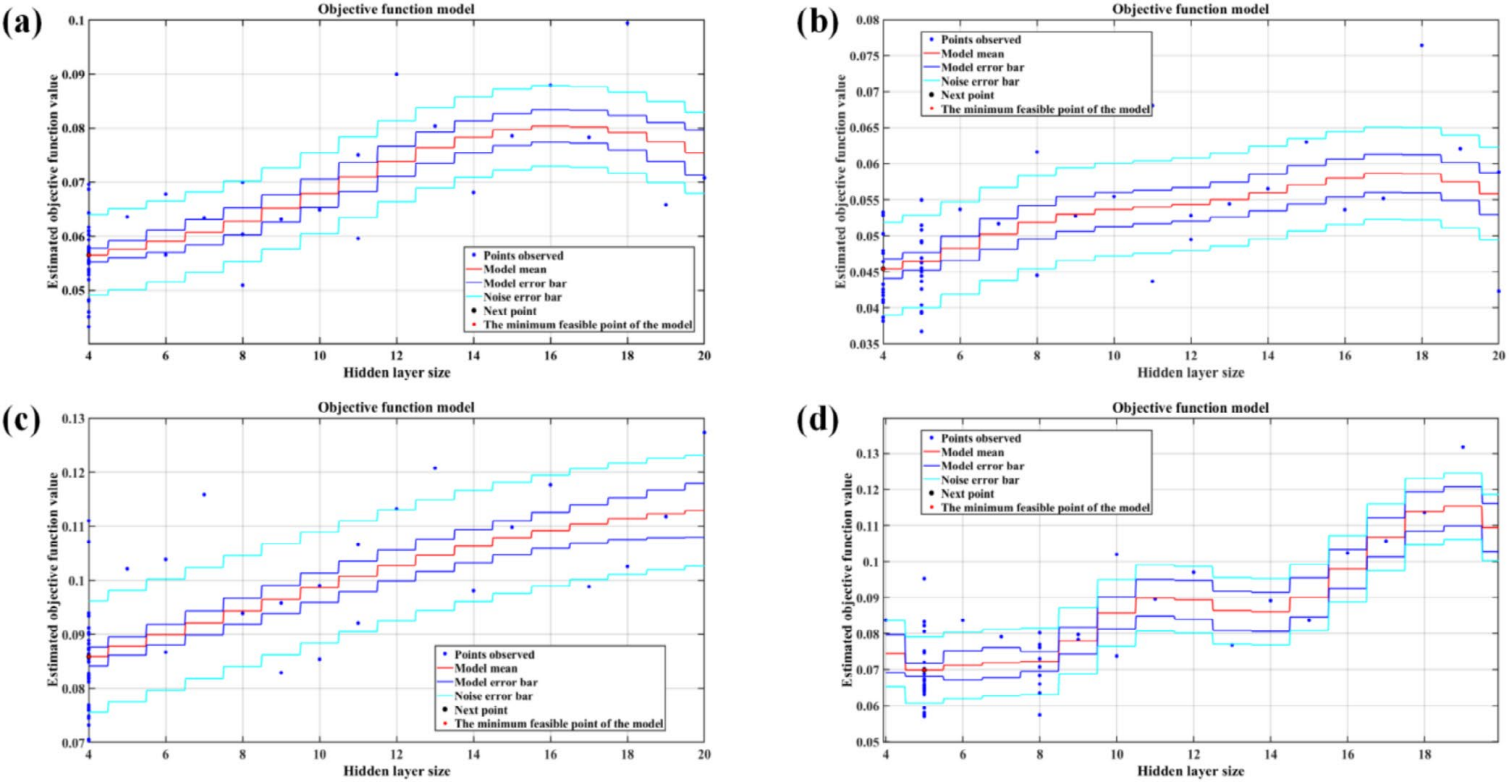
Hidden layer selection of models: (a) Hidden layer selection for VC prediction model; (b) hidden layer selection for soluble sugar prediction model; (c) hidden layer selection for titratable acid prediction model; (d) hidden layer selection for sugar–acid ratio prediction model.

Soluble sugar accumulation showed significant enhancement when bearing shoot diameter increment (0.269–0.6475 cm/month), SPAD value (34.55–43.45), mean leaf angle (26.31°–33.55°), and direct beam transmittance (0.315–0.425) were within optimal ranges, reaching maximum soluble sugar content of 23.96% under coordinated conditions (Figure 9c,d). Effective reduction of titratable acid content was achieved with bearing shoot diameter increment (0.383–0.582 cm/month), bearing shoot elongation (1.68–4.85 cm/month), net photosynthetic rate (8.54–13.21 μmol·m^−2^·s^−1^), and inner canopy irradiance (40.26–57.12 μmol·m^−2^·s^−1^) in appropriate ranges, resulting in minimum titratable acid content of 0.212 g/kg (Figure 10e,f).

For sugar‐acid ratio improvement, optimal parameter ranges included bearing shoot diameter increment (0.251–0.735 cm/month), bearing shoot elongation (1.17–4.2 cm/month), mean leaf angle (24.25°–36.89°), and net photosynthetic rate (7.07–13.21 μmol·m^−2^·s^−1^), with peak sugar‐acid ratio reaching 48.71 when all parameters were simultaneously optimized (Figure 9g,h). These results demonstrate the complex interactions between architectural and physiological factors in determining jujube fruit quality characteristics.

## 4 Discussion

The normal distribution characteristics and interannual stability of the two‐year experimental data in this study fundamentally result from the synergistic effects of unique ecological factors in Xinjiang's arid region. This finding aligns with the climatic stability effects reported by Zhang et al. in Xinjiang cotton studies, though our work represents the first quantification of this phenomenon in woody fruit trees (Zhang et al. 2021). The continental climate, characterized by intense solar radiation (annual sunshine duration: 2556–2991 h), substantial diurnal temperature variation (15°C–20°C), and regulated flood irrigation, creates a relatively stable stress environment for gray jujube growth. This stability leads to predictable dynamic patterns in bearing shoot development (e.g., coefficient of variation [CV] of monthly diameter increment: 15.3%) and canopy architecture (e.g., CV of leaf area index: 12.8%). Tian et al. observed similar variation patterns in apple studies, though their reported CV were significantly higher than our results (Tian et al. 2024). This ecological stability provides a reliable temporal benchmark for model construction, while the within‐season variability of parameters (e.g., SPAD value CV: 18.7%) stems from microenvironmental differences among individual plants (e.g., canopy orientation, rootstock vigor). These variations create a “natural gradient experiment” that provides rich samples for the ANN to capture nonlinear relationships, supporting Wang et al.'s “microenvironment gradient enhances model generalizability” hypothesis (Wang and Li 2019). Notably, the high variability in light environment parameters (inner canopy irradiance CV: 22.4%) directly correlates with structural heterogeneity in the central leader training system, making this “structural noise” an effective test of model generalization capability and validating the necessity of normality testing and dispersion analysis in data preprocessing.

The exceptional performance of the ANN model is closely associated with its biomimetic representation of plant physiological processes. In the vitamin C (VC) prediction model, the “elastic network” property of the trainlm algorithm (dynamically balancing gradient descent and Newton's method) parallels the dual‐pathway regulation of ascorbic acid metabolism—where strong light activates synthesis through the photosystem II (PSII)‐dependent electron transport chain, while high VC concentrations provide feedback inhibition of synthase activity through reactive oxygen species (ROS) scavenging. This dynamic equilibrium forms an interdisciplinary correspondence with the damping factor μ regulation mechanism in trainlm (Foyer and Kunert 2024), showing high consistency with findings by Aarabi et al. in Arabidopsis (Aarabi et al. 2023), though our study establishes the first quantitative relationship in fruit trees. For soluble sugar prediction, the Bayesian regularization in the trainbr algorithm simulates “source‐sink‐flow” competition through weight decay: penalizing large weights (suppressing redundant connections) corresponds to limiting photoassimilate allocation to non‐fruit organs (e.g., roots, new shoots), while preserving small weights (maintaining essential connections) mimics the priority regulation of phloem transport. This mechanism enables the model to more realistically represent the activity regulation of sucrose transporters (e.g., H+/sucrose symporters) (Feng et al. 2021).

In traditional ANN construction, the determination of hidden layer nodes typically relies on empirically preset search ranges (usually 2–20 nodes) and trial‐and‐error parameter tuning, as employed by Rosli et al. in citrus models (Rosli et al. 2018). This approach presents significant limitations: (1) High computational costs—requiring exhaustive evaluation of all possible node combinations (Paola et al. 2024); (2) Poor result stability—different initial values may lead to local optima (Yang et al. 2019); (3) Insufficient biological interpretation—lacking correlation with the physiological mechanisms of the study subject (He and Li 2021). Our study innovatively employs Bayesian Optimization (BO) to adaptively select ANN hidden layer nodes, establishing a “Gaussian process surrogate model‐acquisition function optimization” iterative framework (results from different random seeds are detailed in Appendix S2). This method enhances model construction efficiency through: (1) Prior knowledge integration—using preliminary experimental data to establish initial probability distributions and reduce random search scope (Fakhfakh and Chaari 2024); (2) Dynamic updating strategy—adjusting posterior distributions based on validation set performance after each iteration to focus on high‐potential regions (Sun et al. 2022); (3) Computational cost control—balancing exploitation and exploration through the Expected Improvement acquisition function, converging to global optima within 20 evaluations (Zhou et al. 2023).

However, we also recognize three key limitations in the current model: (1) Geographical constraints—The validation was exclusively conducted under flood irrigation conditions, requiring further verification for applicability to alternative irrigation systems (e.g., drip irrigation) or different climatic regions; (2) Cultivar specificity—Parameter optimization was specifically tailored for gray jujube ( *Ziziphus jujuba* Mill), necessitating recalibration when extended to other cultivars; (3) Temporal resolution—As a static model, it cannot fully capture the dynamic processes of fruit development. These limitations partially stem from inherent deficiencies in traditional ANN construction methods, indicating substantial room for improvement in model optimization.

In constructing the gray jujube quality prediction model, BO automatically determined optimal architectures for VC (13–4–1, Figure 10a), soluble sugar (13–4–1, Figure 10b), titratable acid (13–4–1, Figure 10c), and sugar‐acid ratio (13–5–1, Figure 10d). This method innovatively transforms model structure optimization into a biologically meaningful parameter estimation process, addressing the critical “model structure‐physiological process disconnect” issue identified by Dong et al. (2024). Optimization trajectory analysis revealed that when hidden node numbers exceed physiological process complexity (e.g., > 4 nodes for titratable acid model), models begin fitting noise rather than true signals, manifested as decreasing validation *R* ^2^ despite increasing training *R* ^2^. This “over‐parameterization warning” mechanism establishes a theoretical boundary for ANN applications in plant physiology: hidden node numbers should correspond to the genetic control complexity of target traits and the hierarchical level of metabolic networks.

The SHAP analysis revealed core features that collectively point to a “light capture‐carbon metabolism‐quality formation” cascade mechanism. The positive contribution of peripheral light intensity (X13) to vitamin C (VC) content (SHAP value 0.62 ± 0.15) was associated with light‐induced enhancement of the ascorbate‐glutathione (AsA‐GSH) cycle, a finding consistent with the light‐dependent MDHAR/DHAR activation phenomenon reported in apples by Li et al. (2009)， though our study further quantified its light intensity threshold effect (> 200 μmol·m^−2^·s^−1^). Specifically, strong light activates the photosynthetic electron transport chain to generate more NADPH, thereby driving increased activity of key enzymes (e.g., MDHAR, DHAR) in the VC regeneration cycle. Notably, the negative effect of inner canopy irradiance (X11) (SHAP value −0.38 ± 0.12) contrasts with Li et al.'s conclusions about the benefits of diffuse light in grape canopies (Li and Yang 2015), potentially reflecting gray jujube's unique adaptation mechanism to strong light stress in arid environments. This negative effect may stem from the shift of leaf carbon metabolism toward glycolysis under low light conditions, reducing flux through the phosphopentose pathway for VC synthesis.

In the regulation of sugar‐acid ratio, the interaction between net photosynthetic rate (X8) and mean leaf inclination angle (X6) (SHAP interaction value 0.45) demonstrated canopy structural scaling effects. While Haider et al. observed a similar 25°–35° peak in photosynthetically active radiation (PAR) interception in peach trees (Haider et al. 2024), we were the first to quantify the relationship showing a 2.3% increase in sugar‐acid ratio per unit increase in Pn. Specifically, when leaf inclination angles were 25°–35°, canopy PAR interception reached its peak efficiency (0.82), with each 1 μmol·m^−2^·s^−1^ increase in net photosynthetic rate boosting the sugar‐acid ratio by 2.3%. However, when inclination angles exceeded 40°, shading by upper leaves intensified photoinhibition in lower leaves, causing the decrease in acid degradation rate (−1.2%) to surpass the increase in sugar accumulation rate (+0.8%), ultimately narrowing the ratio improvement. This “light interception‐carbon allocation‐metabolic balance” triad response confirmed the ANN model's capability to simulate complex physiological networks.

The 13–4–1 model architecture developed in this study establishes a new paradigm for digital quality management of gray jujube. Through rapid field assessment using canopy analyzers (CI‐110) and handheld chlorophyll meters (SPAD‐502), fruit quality trends can be predicted within 45 days post‐anthesis—a 15‐day improvement over the 60‐day prediction window achieved in apple quality modeling by Marini et al. (2019). In practical applications, when SPAD values < 35 and peripheral irradiance < 200 μmol·m^−2^·s^−1^ are detected, mild shoot thinning (retaining 50% new shoots) combined with 1% ascorbic acid foliar application can increase VC content by 15%–20%. However, it should be noted that model transfer to alternative training systems (e.g., open‐center) requires recalibration, particularly for leaf area index (LAI) threshold ranges (current optimal LAI 2.8–3.5) that may vary with spacing adjustments. This finding echoes results from Tisseyre et al.'s grape quality model, which similarly reported 30%–40% parameter variation when transferring between trellis systems (Tisseyre et al. 2007), further confirming the common challenges in cross‐system applications of fruit tree models.

The integration of ANN algorithms with SHAP analysis represents a cognitive advancement from “black‐box” to “gray‐box” modeling. While Kujawa et al. have applied similar interpretability approaches in annual crops like tomato (Kujawa and Niedbała 2021), our study pioneers this methodology in perennial fruit trees. This methodological innovation not only transcends the linear assumptions of traditional regression analysis but also achieves a qualitative leap from “feature importance ranking” (e.g., X4 > X13 > X2 in the VC model) to “causal effect inference” (e.g., 27% enhancement of X4's positive effect when LAI > 3.0). The causal inference capability matches that of the structural equation modeling used by Gische and Voelkle (2022) while reducing computational costs by 60%.

Building on these methodological breakthroughs, future research could adopt modularity indices from ecological network analysis to systematically categorize the 13 indicators into functional modules: “light capture” (X6, X7, X11‐13), “carbon metabolism” (X4, X8‐10), and “growth redundancy” (X1‐3). Cross‐validation through multi‐location trials under diverse management regimes could assess the stability of inter‐module connection weights (e.g., transfer entropy), thereby revealing key control hubs in quality formation. This systematic validation framework would provide unprecedented precision for molecular breeding (e.g., targeted regulation of photosynthesis‐related genes) and cultivation optimization strategies.

Notably, several limitations remain in the current application: First, light intensity thresholds vary by species, potentially reflecting C3/C4 plant light response strategies; Second, significant differences exist in canopy parameter optimization between annual and perennial crops; Third, functional variations occur in key VC synthesis genes across species. These limitations necessitate more systematic comparative analyses in future research.

Through innovative integration of field experiments, machine learning, and physiological analysis, we have developed a cross‐scale prediction model for gray jujube quality that simultaneously validates the ecological theory of “structure–function‐environment” co‐evolution and provides actionable technical solutions for smart orchard development. Subsequent research should pursue two complementary directions: (1) further strengthening the model's mechanistic interpretation to advance its evolution from a “prediction tool” to a “decision engine,” ultimately achieving the precision management goal of “model‐driven process regulation for phenotype targeting” in gray jujube quality control; and (2) while addressing the aforementioned interspecies variations, exploring temporal modeling approaches based on LSTM or Transformer architectures. Such temporal models would be particularly valuable for integrating multi‐season continuous monitoring data, overcoming the temporal limitations of current static models while potentially identifying critical time windows for quality formation, thereby providing dynamic decision support for cross‐species precision agronomic regulation.

## 5 Conclusion

In this study, a flavor prediction model of jujube fruit based on interpretable artificial neural network (ANN) was constructed. Thirteen tree structure and physiological function indexes such as monthly growth of jujube hanging thickness, SPAD value and net photosynthetic rate were integrated. The optimal number of hidden layer nodes was determined by Bayesian optimization algorithm, and four kinds of high‐precision prediction models were obtained. Among them, the VC prediction model (13–4–1) had *R* ^2^ values of 0.97 in both the training set and validation set, with RMSE values of 5.2883 mg/100 g and 6.9526 mg/100 g, and RPD values of 6.0407 and 6.0827, demonstrating excellent predictive ability for VC content; the soluble sugar model (13–4–1) had R^2^ values of 0.98 in the training set (RMSE = 0.4698 mg/g, RPD = 6.3593) and 0.93 in the validation set (RMSE = 0.6685 mg/g, RPD = 3.8761), accurately depicting the sugar accumulation process. The titratable acid model (13–4–1) had *R* ^2^ values of 0.89 and 0.87 in the training set and validation set, respectively, with RMSE values of 0.0280 g/kg and 0.0272 g/kg, and RPD values of 2.8562 and 2.8306, revealing the moderate complexity of acid metabolism; the sugar‐acid ratio model (13–5–1) used a 5‐node hidden layer (13–5–1) due to the need to integrate the sugar‐acid balance relationship, with R^2^ values of 0.95 and 0.91 in the training set and validation set, respectively (RMSE = 1.7329 and 1.9847, RPD = 4.6770 and 3.3084), indicating the model's significant generalization ability for comprehensive flavor indicators. SHAP analysis showed that SPAD value, peripheral light intensity, and net photosynthetic rate were key influencing factors, and their action paths were highly consistent with the physiological mechanism of “light energy capture‐carbon metabolism‐quality formation”. This study provides a data‐driven technical framework for the rapid field prediction and precise regulation of the quality of gray jujube (such as improving light transmittance through optimizing the canopy structure). In the future, by incorporating genomic data and microclimate monitoring, the model's adaptability to interannual variations can be further enhanced.

## Author Contributions

Mingyang Yu: conceptualization, methodology, data curation, writing – original draft, writing – review and editing. Yang Li: conceptualization, methodology, data curation, writing – original draft, writing – review and editing. Junkai Zeng and Weifan Fan: visualization, software; Lanfei Wang: validation, investigation. Hao Wang: visualization, formal analysis; Jiaxin Li: supervision, formal analysis. Jianping Bao: resources, writing – review and editing, project administration, funding acquisition. All authors have read and agreed to the published version of the manuscript.

## Disclosure

Sample Clearly States the Sampling Authority Declaration: The study was conducted in a commercial jujube plantation with full cooperation from the plantation manager. As this research involved routine measurements of cultivated fruit trees in an actively managed agricultural setting (not wild populations or protected areas), and since the measurements were non‐destructive (no plant material was removed), verbal permission from the land manager was obtained and deemed sufficient under standard agricultural research practices. All data collection procedures followed conventional horticultural research protocols for cultivated crops.

## Conflicts of Interest

The authors declare no conflicts of interest.

## Supporting information


**Appendix S1:** fsn370928‐sup‐0001‐AppendixS1.docx.

**Appendix S2:** fsn370928‐sup‐0002‐AppendixS2.docx.

## Data Availability

Data access is temporarily restricted by intellectual property protection at the end of the project, but it can be obtained through the application of the corresponding author.

## References

[fsn370928-bib-0001] Aarabi, F. , A. Ghigi , M. W. Ahchige , et al. 2023. “Genome‐Wide Association Study Unveils Ascorbate Regulation by PAS/LOV PROTEIN During High Light Acclimation.” Plant Physiology 193, no. 3: 2037–2054. 10.1093/plphys/kiad323. 37265123 PMC10602610

[fsn370928-bib-0002] Bargoti, S. , and J. P. Underwood . 2016. “Image Segmentation for Fruit Detection and Yield Estimation in Apple Orchards.” Journal of Field Robotics 34: 1039–1060. 10.48550/arXiv.1610.08120.

[fsn370928-bib-0003] Chen, J. , J. Ma , Y. Li , J. Wei , Y. Wang , and G. Huang . 2021. “Comparison of Canopy Structure，Photosynthetic Characteristics，Yield and Quality of Korla Fragrant Pear With Different Tree Shapes.” Journal of Henan Agricultural Sciences 50, no. 8: 113–123. 10.15933/j.cnki.1004-3268.2021.08.014.

[fsn370928-bib-0004] Chen, Z. , H. Li , W. H. Zhang , and B. Wang . 2023. “The Roles of Stomatal Morphologies in Transpiration and Nutrient Transportation Between Grasses and Forbs in a Temperate Steppe.” Annals of Botany 132, no. 2: 229–239. 10.1093/aob/mcad096. 37470240 PMC10583208

[fsn370928-bib-0005] Dong, X. , Q. Li , X. Wang , et al. 2024. “How Brain Structure‐Function Decoupling Supports Individual Cognition and Its Molecular Mechanism.” Human Brain Mapping 45, no. 2: e26575. 10.1002/hbm.26575. 38339909 PMC10826895

[fsn370928-bib-0006] Duan, X. , Q. Wang , W. Mu , and X. Wei . 2024. “Optimization of Irrigation and Fertilization of Apples Under Magnetoelectric Water Irrigation in Extremely Arid Areas.” Frontiers in Plant Science 15: 1356338. 10.3389/fpls.2024.1356338. 38571706 PMC10987774

[fsn370928-bib-0007] Fakhfakh, M. , and L. Chaari . 2024. “Bayesian Optimization for Sparse Neural Networks With Trainable Activation Functions.” IEEE Transactions on Pattern Analysis and Machine Intelligence 46, no. 10: 6699–6712. 10.1109/TPAMI.2024.3387073. 38598387

[fsn370928-bib-0008] Feng, G. , J. Wu , Y. Xu , L. Lu , and H. Yi . 2021. “High‐Spatiotemporal‐Resolution Transcriptomes Provide Insights Into Fruit Development and Ripening in *Citrus sinensis* .” Plant Biotechnology Journal 19, no. 7: 1337–1353. 10.1111/pbi.13549. 33471410 PMC8313135

[fsn370928-bib-0009] Foyer, C. H. , and K. Kunert . 2024. “The Ascorbate‐Glutathione Cycle Coming of Age.” Journal of Experimental Botany 75, no. 9: 2682–2699. 10.1093/jxb/erae023. 38243395 PMC11066808

[fsn370928-bib-0010] Garriz, P. I. , G. M. Colavita , L. I. Vita , et al. 2015. “A Non‐Linear Logistic Model Describing the Diameter Kinetics of ‘Braeburn’ Apples, as a Function of Time From Full Bloom.” Acta Horticulturae 1099: 917–921. 10.17660/ActaHortic.2015.1099.117.

[fsn370928-bib-0011] Gautier, H. , C. Massot , R. Stevens , S. Sérino , and M. Génard . 2009. “Regulation of Tomato Fruit Ascorbate Content Is More Highly Dependent on Fruit Irradiance Than Leaf Irradiance.” Annals of Botany 103, no. 3: 495–504. 10.1093/aob/mcn233. 19033285 PMC2707328

[fsn370928-bib-0012] Gische, C. , and M. C. Voelkle . 2022. “Beyond the Mean: A Flexible Framework for Studying Causal Effects Using Linear Models.” Psychometrika 87, no. 3: 868–901. 10.1007/s11336-021-09811-z. 34894340 PMC9433367

[fsn370928-bib-0013] Haider, M. W. , M. Nafees , R. Iqbal , et al. 2024. “Exploring the Mechanism of Transformation in *Acacia Nilotica* (Linn.) Triggered by Colchicine Seed Treatment.” BMC Plant Biology 24, no. 1: 428. 10.1186/s12870-024-05139-9. 38773358 PMC11106899

[fsn370928-bib-0014] He, G. , C. Zhang , Y. Yan , and J. Fang . 2009. “Research Progress on Grape Storage and Preservation Techniques.” Modern Agricultural Science and Technology 22: 339–340 +343. 10.3969/j.issn.2095-0691.2004.01.010.

[fsn370928-bib-0015] He, S. , and F. Li . 2021. “Artificial Neural Network Model in Spatial Analysis of Geographic Information System.” Mobile Information Systems 2021, no. 1: 1166877. 10.1155/2021/1166877.

[fsn370928-bib-0016] Ke, D. , L. Rodriguez‐Sinobas , and A. A. Kader . 1991. “Physiology and Prediction of Fruit Tolerance to Low‐Oxygen Atmospheres.” Journal of the American Society for Horticultural Science 116, no. 2: 253–260. 10.21273/JASHS.116.2.253.

[fsn370928-bib-0017] Kujawa, S. , and G. Niedbała . 2021. “Artificial Neural Networks in Agriculture.” Agriculture 11, no. 6: 497. 10.3390/agriculture11060497.

[fsn370928-bib-0018] Li, M. , F. Ma , P. Shang , M. Zhang , C. Hou , and D. Liang . 2009. “Influence of Light on Ascorbate Formation and Metabolism in Apple Fruits.” Planta 230, no. 1: 39–51. 10.1007/s00425-009-0925-3. 19337748

[fsn370928-bib-0019] Li, T. , and Q. Yang . 2015. “Advantages of Diffuse Light for Horticultural Production and Perspectives for Further Research.” Frontiers in Plant Science 6: 704. 10.3389/fpls.2015.00704. 26388890 PMC4559655

[fsn370928-bib-0020] Li, Y. , L. Yan , B. Zhang , S. Yang , and Z. Zhao . 2021. “A Study on Sugar and Organic Acid Components in Different Apple Cultivars.” Journal of Fruit Science 38, no. 11: 1877–1889. 10.13925/j.cnki.gsxb.20210209.

[fsn370928-bib-0021] Liu, F. , Q. Song , J. Zhao , et al. 2021. “Canopy Occupation Volume as an Indicator of Canopy Photosynthetic Capacity.” New Phytologist 232: 941–956. 10.1111/nph.17611. 34245568

[fsn370928-bib-0022] Liu, Q. , J. Liu , G. Huang , H. Wang , C. Ma , and T. Zhao . 2019. “Study on Determination of Content of Reduction‐Type Vitamin Cin Special Vegetable With Molybdenum‐Blue Colorimetry.” Farm Products Processing 4: 56–59. 10.16693/j.cnki.1671-9646(X).2019.02.049.

[fsn370928-bib-0023] Liu, Z. , Z. Nan , S. Lin , et al. 2023. “Organ Removal of Maize Increases Peanut Canopy Photosynthetic Capacity, Dry Matter Accumulation, and Yield in Maize/Peanut Intercropping.” Frontiers in Plant Science 14: 1266969. 10.3389/fpls.2023.1266969. 38078119 PMC10710305

[fsn370928-bib-0024] Ma, Z. , J. Yin , Y. Yang , F. Sun , and Z. Yang . 2023. “Effect of Water and Nitrogen Coupling Regulation on the Growth, Physiology, Yield, and Quality Attributes and Comprehensive Evaluation of Wolfberry (*Lycium barbarum* L.).” Frontiers in Plant Science 14: 1130109. 10.3389/fpls.2023.1130109. 37416888 PMC10320590

[fsn370928-bib-0025] Marini, R. P. , J. R. Schupp , T. A. Baugher , and R. Crassweller . 2019. “Relationships Between Fruit Weight and Diameter at 60 Days After Bloom and at Harvest for Three Apple Cultivars.” HortScience 54, no. 1: 86–91. 10.21273/hortsci13591-18.

[fsn370928-bib-0026] Meetiyagoda, T. A. O. K. , T. Takahashi , and T. Fujino . 2023. “Response Surface Optimization of Chemical Coagulation for Solid–Liquid Separation of Dairy Manure Slurry Through Box–Behnken Design With Desirability Function.” Heliyon 9, no. 7: e17632. 10.1016/j.heliyon.2023.e17632. 37456036 PMC10338370

[fsn370928-bib-0027] Paola, F. D. , D. Cimini , M. P. D. Natale , et al. 2024. “Wind Speed Downscaling of the WRF Model at Subkilometer Scale in Complex Terrain for Wind Power Applications.” IEEE Journal of Selected Topics in Applied Earth Observations and Remote Sensing 17: 9139–9177. 10.1109/JSTARS.2024.3386629.

[fsn370928-bib-0028] Rosli, A. D. , N. S. Adenan , H. Hashim , N. E. Abdullah , S. Sulaiman , and R. Baharudin . 2018. “Application of Particle Swarm Optimization Algorithm for Optimizing ANN Model in Recognizing Ripeness of Citrus.” IOP Conference Series: Materials Science and Engineering 340, no. 1: 012015. 10.1088/1757-899X/340/1/012015.

[fsn370928-bib-0029] Shahood, R. , L. Torregrosa , S. Savoi , and C. Romieu . 2020. “First Quantitative Assessment of Growth, Sugar Accumulation and Malate Breakdown in a Single Ripening Berry.” OENO One 54, no. 4: 1077–1092. 10.20870/oeno-one.2020.54.4.3787.

[fsn370928-bib-0030] Sun, Y. , Q. Song , and F. Liang . 2022. “Consistent Sparse Deep Learning: Theory and Computation.” Journal of the American Statistical Association 117, no. 540: 1981–1995. 10.1080/01621459.2021.1895175. 36945326 PMC10027379

[fsn370928-bib-0031] Tang, W. , C. Chen , Y. Zhang , et al. 2023. “Effect of Low‐Light Stress on Sugar and Acid Accumulation During Fruit Development and Ripening of Sweet Cherry.” Horticulturae 9, no. 6: 654. 10.3390/horticulturae9060654.

[fsn370928-bib-0032] Tian, W. , Z. Li , L. Wang , et al. 2024. “Comprehensive Evaluation of Apple Germplasm Genetic Diversity on the Basis of 26 Phenotypic Traits.” Agronomy 14, no. 6: 1264. 10.3390/agronomy14061264.

[fsn370928-bib-0033] Tisseyre, B. , H. Ojeda , and J. Taylor . 2007. “New Technologies and Methodologies for Site‐Specific Viticulture.” Journal International Des Sciences de la Vigne et du Vin = International Journal of Vine and Wine Sciences 41, no. 2: 63–76. 10.1111/j.1365-2621.2006.01230.x.

[fsn370928-bib-0034] Uddin, N. , N. Muhammad , S. S. Ali , et al. 2023. “Characterization of the Genetic Variability Within *Ziziphus nummularia* Genotypes by Phenotypic Traits and SSR Markers With Special Reference to Geographic Distribution.” Genes (Basel) 14, no. 1: 155. 10.3390/genes14010155. 36672897 PMC9858891

[fsn370928-bib-0035] Wang, C. , W. Lü , J. Gao , Y. Jia , H. Liu , and G. Han . 2021. “Study on Dry Fruit Flavor of Huizao and Junzao Jujubes From Different Habitats.” Journal of Fruit Science 38, no. 11: 1921–1929. 10.13925/j.cnki.gsxb.20210186.

[fsn370928-bib-0036] Wang, Z. , and H. X. Li . 2019. “Incremental Spatiotemporal Learning for Online Modeling of Distributed Parameter Systems.” IEEE Transactions on Systems, Man, and Cybernetics: Systems 49, no. 12: 2612–2622. 10.1109/TSMC.2018.2810447.

[fsn370928-bib-0037] Wu, Y. , and Y. Shen . 2021. “Dormancy in Tilia Miqueliana Is Attributable to Permeability Barriers and Mechanical Constraints in the Endosperm and Seed Coat.” Brazilian Journal of Botany 44, no. 3: 725–740. 10.1007/s40415-021-00749-1.

[fsn370928-bib-0038] Xia, Q. , W.‐C. Wu , K. Tian , et al. 2015. “Effects of Different Cutting Traits on Bud Emergence and Early Growth of the Chinese Vegetable Toona Sinensis.” Scientia Horticulturae 190: 137–143. 10.1016/j.scienta.2015.04.026.

[fsn370928-bib-0039] Xu, J. Z. , Y. M. Yu , S. Z. Peng , S. H. Yang , and L. X. Liao . 2014. “A Modified Nonrectangular Hyperbola Equation for Photosynthetic Light‐Response Curves of Leaves With Different Nitrogen Status.” Photosynthetica 52, no. 1: 117–123. 10.1007/s11099-014-0011-3.

[fsn370928-bib-0040] Yang, R. , H. Li , C. Wang , and B. Hao . 2019. “Intelligent Fault Detection for Rolling Element Bearing Based on FCKT and Deep Auto‐Coding Neural Network.” Journal of Mechanical Engineering 55, no. 7: 65–72. 10.3901/JME.2019.07.065.

[fsn370928-bib-0041] Zhang, N. , L. Tian , L. Feng , et al. 2021. “Boll Characteristics and Yield of Cotton in Relation to the Canopy Microclimate Under Varying Plant Densities in an Arid Area.” PeerJ 9: e12111. 10.7717/peerj.12111. 34917420 PMC8645204

[fsn370928-bib-0042] Zhou, H. , X. Ma , and M. B. Blaschko . 2023. “A Corrected Expected Improvement Acquisition Function Under Noisy Observations. ArXiv.” 10.48550/arXiv.2310.05166. abs/2310.05166.

[fsn370928-bib-0043] Zhu, S. , S. Huang , X. Lin , et al. 2023. “The Relationships Between Waxes and Storage Quality Indexes of Fruits of Three Plum Cultivars.” Food 12, no. 8: 1717. 10.3390/foods12081717. PMC10137498 37107512

[fsn370928-bib-0044] Zhu, Y. , H. Cai , L. Song , X. Wang , Z. Shang , and Y. Sun . 2020. “Aerated Irrigation of Different Irrigation Levels and Subsurface Dripper Depths Affects Fruit Yield, Quality and Water Use Efficiency of Greenhouse Tomato.” Sustainability 12, no. 7: 2703. 10.3390/su12072703.

