# Supplementary material for: Mechanism and Prediction of Gray Jujube Fruit Quality Using Explainable ANN
Source: Food Sci Nutr. 2025 Sep 16;13(9):e70928. doi: 10.1002/fsn3.70928 (PMC12438962; doi:10.1002/fsn3.70928)
Supplement: Supplementary file 1 — Appendix S1: fsn370928‐sup‐0001‐AppendixS1.docx. [file FSN3-13-e70928-s001.docx]

**TABLE 1:** Grey dates VC model indicators based on ANN algorithm.

| Training function | Training function | Connection function | R2 | RMSE | MSE | RPD | MAE | MBE | MAPE |
| --- | --- | --- | --- | --- | --- | --- | --- | --- | --- |
| traingdx | radbas-tansig | Training set | 0.97204 | 5.88560 | 34.64070 | 6.05050 | 4.22700 | 0.89678 | 0.05399 |
|  |  | Validation set | 0.77653 | 15.33150 | 235.05520 | 2.15320 | 10.19880 | 2.85950 | 0.12200 |
| traingdx | radbas-logsig | Training set | 0.28139 | 29.03510 | 843.03950 | 1.49240 | 21.24040 | 17.78590 | 0.42748 |
|  |  | Validation set | 0.28269 | 29.40820 | 864.83960 | 1.51090 | 22.72390 | 18.34880 | 0.41551 |
| traingdx | radbas-poslin | Training set | 0.05159 | 33.41290 | 1116.42400 | 1.09970 | 28.38300 | 11.95960 | 0.49689 |
|  |  | Validation set | 0.08580 | 32.86740 | 1080.26410 | 1.07000 | 26.94690 | 6.94500 | 0.41693 |
| traingdx | radbas-purelin | Training set | 0.96554 | 6.19900 | 38.42770 | 5.38670 | 4.58480 | 0.01603 | 0.06859 |
|  |  | Validation set | 0.89313 | 11.97720 | 143.45420 | 3.11080 | 7.47680 | 2.17940 | 0.10182 |
| traingdx | logsig-tansig | Training set | 0.53674 | 22.74710 | 517.43110 | 1.47790 | 18.48510 | 2.46400 | 0.27065 |
|  |  | Validation set | 0.31218 | 28.90730 | 835.63020 | 1.23110 | 23.34400 | 5.83440 | 0.27122 |
| traingdx | logsig-logsig | Training set | —— | | | | | | |
|  |  | Validation set |  |  |  |  |  |  |  |
| traingdx | logsig-poslin | Training set | —— | | | | | | |
|  |  | Validation set |  |  |  |  |  |  |  |
| traingdx | logsig-Purelin | Training set | 0.90944 | 10.45050 | 109.21290 | 3.32550 | 7.53150 | 0.40125 | 0.10104 |
|  |  | Validation set | 0.87738 | 11.76000 | 138.29780 | 2.86950 | 8.60830 | 1.14940 | 0.14277 |
| traingdx | Tansing-tansig | Training set | 0.96330 | 6.24710 | 39.02650 | 5.22020 | 4.66620 | 0.01470 | 0.05598 |
|  |  | Validation set | 0.92874 | 9.95770 | 99.15660 | 3.74870 | 6.29140 | 0.38129 | 0.09694 |
| traingdx | Tansig-logsig | Training set | 0.36597 | 25.46190 | 648.31020 | 1.59300 | 18.64880 | 15.66430 | 0.36335 |
|  |  | Validation set | 0.42345 | 29.96580 | 897.95120 | 1.49410 | 22.74380 | 14.15150 | 0.44019 |
| traingdx | Tansig-poslin | Training set | —— | | | | | | |
|  |  | Validation set |  |  |  |  |  |  |  |
| traingdx | Tansig-Purelin | Training set | 0.61492 | 21.62740 | 467.74370 | 1.61150 | 17.35900 | 0.16304 | 0.23731 |
|  |  | Validation set | 0.39054 | 25.79670 | 665.47110 | 1.28490 | 21.66040 | 2.03010 | 0.36094 |
| traingdx | Purelin-tansig | Training set | 0.69142 | 19.04390 | 362.67160 | 1.80420 | 15.92760 | 1.26850 | 0.21144 |
|  |  | Validation set | 0.75422 | 16.62980 | 276.55070 | 2.10960 | 13.29400 | 4.87050 | 0.20933 |
| traingdx | Purelin-logsig | Training set | 0.10183 | 32.26480 | 1041.01620 | 1.30120 | 26.42200 | 18.88050 | 0.49751 |
|  |  | Validation set | 0.36573 | 27.50540 | 756.54890 | 1.43870 | 21.24550 | 13.42590 | 0.34457 |
| traingdx | Purelin-poslin | Training set | 0.30813 | 27.60070 | 761.79800 | 1.53420 | 20.00280 | 17.14640 | 0.39071 |
|  |  | Validation set | 0.24998 | 31.94610 | 1020.55370 | 1.51660 | 24.78800 | 20.71170 | 0.49155 |
| traingdx | Purelin-Purelin | Training set | 0.95670 | 6.55590 | 42.97920 | 4.82310 | 5.23650 | 0.55578 | 0.08200 |
|  |  | Validation set | 0.94792 | 8.84390 | 78.21420 | 4.77470 | 6.06040 | 3.51170 | 0.08252 |
| traingdx | poslin-tansig | Training set | 0.35513 | 27.55370 | 759.20870 | 1.24540 | 22.67880 | 0.43073 | 0.27652 |
|  |  | Validation set | 0.18558 | 31.20360 | 973.66480 | 1.13900 | 25.64980 | 7.22360 | 0.31616 |
| traingdx | poslin-logsig | Training set | —— | | | | | | |
|  |  | Validation set |  |  |  |  |  |  |  |
| traingdx | poslin-poslin | Training set | —— | | | | | | |
|  |  | Validation set |  |  |  |  |  |  |  |
| traingdx | poslin-Purelin | Training set | 0.90792 | 10.66800 | 113.80710 | 3.29850 | 7.57520 | 0.45669 | 0.10331 |
|  |  | Validation set | 0.89900 | 10.23870 | 104.83070 | 3.23980 | 7.85000 | 2.43980 | 0.11404 |
| Training function | Training function | Connection function | R2 | RMSE | MSE | RPD | MAE | MBE | MAPE |
| trainbr | radbas-tansig | Training set | 0.97194 | 5.75400 | 33.10880 | 5.98530 | 2.95220 | 0.41776 | 0.03579 |
|  |  | Validation set | 0.94285 | 8.02770 | 64.44390 | 4.27310 | 5.47500 | 1.64080 | 0.07448 |
| trainbr | radbas-logsig | Training set | 0.16513 | 30.31540 | 919.02300 | 1.43440 | 22.94720 | 19.59660 | 0.45208 |
|  |  | Validation set | 0.37603 | 28.56150 | 815.75920 | 1.40180 | 19.73670 | 12.26520 | 0.37355 |
| trainbr | radbas-poslin | Training set | —— | | | | | | |
|  |  | Validation set |  |  |  |  |  |  |  |
| trainbr | radbas-purelin | Training set | 0.96231 | 6.79310 | 46.14570 | 5.15150 | 3.10720 | 0.10853 | 0.04315 |
|  |  | Validation set | 0.90483 | 10.07020 | 101.40930 | 3.36330 | 6.54610 | 2.68610 | 0.10008 |
| trainbr | logsig-tansig | Training set | 0.98405 | 4.29720 | 18.46590 | 7.92030 | 2.56700 | 0.11490 | 0.09637 |
|  |  | Validation set | 0.87854 | 12.28340 | 150.88130 | 3.07450 | 8.30220 | 4.41140 | 0.09638 |
| trainbr | logsig-logsig | Training set | 0.33483 | 27.49980 | 756.23780 | 1.52510 | 19.22410 | 16.35270 | 0.38042 |
|  |  | Validation set | 0.07651 | 33.46830 | 1120.12690 | 1.40580 | 26.28200 | 22.50210 | 0.51854 |
| trainbr | logsig-poslin | Training set | —— | | | | | | |
|  |  | Validation set |  |  |  |  |  |  |  |
| trainbr | logsig-Purelin | Training set | 0.97592 | 5.01420 | 25.14240 | 6.50680 | 2.93550 | 0.69508 | 0.00337 |
|  |  | Validation set | 0.94616 | 7.66190 | 58.70440 | 4.31500 | 5.63180 | 0.37403 | 0.07975 |
| trainbr | Tansing-tansig | Training set | 0.98869 | 3.62270 | 13.12430 | 9.48160 | 2.02480 | 0.46026 | 0.02659 |
|  |  | Validation set | 0.91491 | 10.22060 | 104.46140 | 3.54870 | 5.51700 | 2.63990 | 0.05988 |
| trainbr | Tansig-logsig | Training set | —— | | | | | | |
|  |  | Validation set |  |  |  |  |  |  |  |
| trainbr | Tansig-poslin | Training set | —— | | | | | | |
|  |  | Validation set |  |  |  |  |  |  |  |
| trainbr | Tansig-Purelin | Training set | 0.98961 | 3.35880 | 11.28120 | 9.80870 | 1.92800 | 0.02419 | 0.02837 |
|  |  | Validation set | 0.85441 | 14.02120 | 196.59540 | 2.69810 | 10.09400 | 3.33190 | 0.12998 |
| trainbr | Purelin-tansig | Training set | 0.97628 | 5.07230 | 25.72780 | 6.56350 | 3.41440 | 0.73804 | 0.04112 |
|  |  | Validation set | 0.93766 | 9.34280 | 87.28730 | 4.01750 | 4.62090 | 0.73413 | 0.05257 |
| trainbr | Purelin-logsig | Training set | 0.29327 | 27.89840 | 778.32150 | 1.50220 | 20.83730 | 17.03850 | 0.42102 |
|  |  | Validation set | 0.48378 | 26.39970 | 696.94370 | 1.56120 | 20.01770 | 11.95820 | 0.35173 |
| trainbr | Purelin-poslin | Training set | —— | | | | | | |
|  |  | Validation set |  |  |  |  |  |  |  |
| trainbr | Purelin-Purelin | Training set | 0.97505 | 5.71200 | 32.62640 | 6.33050 | 3.91900 | 0.04262 | 0.05009 |
|  |  | Validation set | 0.95925 | 5.99610 | 35.95350 | 5.04900 | 4.05390 | 1.15880 | 0.05440 |
| trainbr | poslin-tansig | Training set | 0.95421 | 7.38910 | 54.59930 | 4.75120 | 3.52000 | 1.33430 | 0.03690 |
|  |  | Validation set | 0.97631 | 5.11710 | 26.18460 | 6.51050 | 3.37510 | 0.33308 | 0.04428 |
| trainbr | poslin-logsig | Training set | 0.22799 | 29.24970 | 855.54210 | 1.44480 | 21.52900 | 18.01910 | 0.43305 |
|  |  | Validation set | 0.36850 | 29.21454 | 853.53900 | 1.46510 | 21.75320 | 14.96270 | 0.40155 |
| trainbr | poslin-poslin | Training set | —— | | | | | | |
|  |  | Validation set |  |  |  |  |  |  |  |
| trainbr | poslin-Purelin | Training set | 0.97976 | 4.75960 | 22.65330 | 7.02980 | 2.74800 | 0.05537 | 0.03802 |
|  |  | Validation set | 0.95056 | 8.00050 | 64.00730 | 4.55930 | 5.46890 | 1.31600 | 0.09199 |

| Training function | Training function | Connection function | R2 | RMSE | MSE | RPD | MAE | MBE | MAPE |
| --- | --- | --- | --- | --- | --- | --- | --- | --- | --- |
| trainlm | radbas-tansig | Training set | 0.92854 | 8.98410 | 80.71440 | 3.79520 | 4.37890 | 1.51490 | 0.05086 |
|  |  | Validation set | 0.94252 | 8.44310 | 5.52350 | 4.34070 | 5.52350 | 1.33690 | 0.06087 |
| trainlm | radbas-logsig | Training set | 0.23553 | 28.64580 | 820.58080 | 1.48700 | 24.60190 | 15.79060 | 0.43495 |
|  |  | Validation set | 0.21446 | 29.76840 | 921.97520 | 1.56780 | 25.97670 | 14.98700 | 0.45312 |
| trainlm | radbas-poslin | Training set | 0.59612 | 44.32890 | 1965.05580 | 1.11930 | 35.63650 | 31.34310 | 0.65803 |
|  |  | Validation set | 0.58259 | 45.60950 | 2080.22350 | 1.06490 | 36.41570 | 33.84580 | 0.68421 |
| trainlm | radbas-purelin | Training set | 0.97816 | 5.27680 | 27.84090 | 6.77930 | 3.61330 | 0.32153 | 0.04728 |
|  |  | Validation set | 0.92391 | 8.52050 | 72.59960 | 3.76360 | 6.09510 | 2.28990 | 0.09019 |
| trainlm | logsig-tansig | Training set | 0.96734 | 6.45610 | 41.68170 | 5.62450 | 3.94930 | 1.15570 | 0.05150 |
|  |  | Validation set | 0.95292 | 6.65510 | 44.29070 | 5.21300 | 5.01540 | 3.11030 | 0.06672 |
| trainlm | logsig-logsig | Training set | 0.30662 | 27.27080 | 743.69880 | 1.49840 | 19.46300 | 16.30960 | 0.40310 |
|  |  | Validation set | 0.46246 | 27.77380 | 771.38280 | 1.59910 | 21.92540 | 14.49800 | 0.37536 |
| trainlm | logsig-poslin | Training set | 0.69348 | 44.09470 | 1944.34070 | 1.18630 | 34.84520 | 33.59360 | 0.64784 |
|  |  | Validation set | 0.62590 | 45.34300 | 2055.98810 | 1.19180 | 35.68240 | 34.14300 | 0.68984 |
| trainlm | logsig-Purelin | Training set | 0.97232 | 5.28830 | 27.96600 | 6.04070 | 3.85110 | 0.52508 | 0.04924 |
|  |  | Validation set | 0.96853 | 6.95260 | 48.33900 | 6.08270 | 4.93990 | 2.61310 | 0.05621 |
| trainlm | Tansing-tansig | Training set | 0.86246 | 12.32320 | 105.32320 | 2.68910 | 9.54770 | 0.47039 | 0.14295 |
|  |  | Validation set | 0.77747 | 19.14550 | 126.14550 | 2.28060 | 12.27950 | 1.30070 | 0.25529 |
| trainlm | Tansig-logsig | Training set | 0.60294 | 44.43770 | 1974.71160 | 1.22330 | 34.69670 | 33.93260 | 0.64354 |
|  |  | Validation set | 0.50219 | 46.30550 | 2144.20310 | 1.05860 | 35.92620 | 34.60930 | 0.72259 |
| trainlm | Tansig-poslin | Training set | 0.32535 | 26.27230 | 690.23200 | 1.54790 | 17.64530 | 16.22500 | 0.35849 |
|  |  | Validation set | 0.42628 | 29.89000 | 893.40950 | 1.77710 | 22.11530 | 20.00850 | 0.46483 |
| trainlm | Tansig-Purelin | Training set | 0.96794 | 6.21890 | 38.67490 | 5.58600 | 4.97610 | 0.11572 | 0.06005 |
|  |  | Validation set | 0.92320 | 9.29070 | 86.31740 | 3.61070 | 7.13760 | 0.33880 | 0.09372 |
| trainlm | Purelin-tansig | Training set | 0.98233 | 4.64050 | 21.53430 | 7.55250 | 2.98770 | 0.40502 | 0.03691 |
|  |  | Validation set | 0.92902 | 8.65740 | 74.95070 | 3.79730 | 4.29370 | 1.31220 | 0.04502 |
| trainlm | Purelin-logsig | Training set | 0.27420 | 28.10450 | 789.86430 | 1.49030 | 19.87510 | 17.31600 | 0.40038 |
|  |  | Validation set | 0.33217 | 30.63390 | 938.43730 | 1.55150 | 22.96580 | 18.83390 | 0.45413 |
| trainlm | Purelin-poslin | Training set | 0.20992 | 29.53090 | 872.09360 | 1.50210 | 20.60670 | 19.56780 | 0.43518 |
|  |  | Validation set | 0.48981 | 26.08890 | 680.63040 | 1.76420 | 17.93240 | 15.87450 | 0.34347 |
| trainlm | Purelin-Purelin | Training set | 0.97071 | 5.96550 | 35.58710 | 5.84730 | 4.28690 | 0.23015 | 0.05457 |
|  |  | Validation set | 0.96704 | 5.95860 | 35.50470 | 5.53850 | 4.38630 | 0.62307 | 0.06019 |
| trainlm | poslin-tansig | Training set | 0.86479 | 13.33990 | 177.95340 | 2.72660 | 9.03970 | 0.95919 | 0.09930 |
|  |  | Validation set | 0.71397 | 15.68450 | 246.00420 | 1.87420 | 9.03970 | 1.07720 | 0.11749 |
| trainlm | poslin-logsig | Training set | 0.28982 | 28.19040 | 794.69840 | 1.55460 | 19.75740 | 18.21170 | 0.40416 |
|  |  | Validation set | 0.29708 | 30.60690 | 936.78490 | 1.49410 | 23.44400 | 18.43210 | 0.44531 |
| trainlm | poslin-poslin | Training set | —— | | | | | | |
|  |  | Validation set |  |  |  |  |  |  |  |
| trainlm | poslin-Purelin | Training set | 0.94727 | 7.70140 | 59.31230 | 4.35510 | 4.20700 | 0.48478 | 0.06479 |
|  |  | Validation set | 0.85264 | 13.26930 | 176.07440 | 2.67380 | 7.56530 | 2.99200 | 0.16494 |

**Note:** The dashes indicate invalid entries.

**TABLE 2:** Model indicators of soluble sugar in jujube based on ANN algorithm.

| Training function | Training function | Connection function | R2 | RMSE | MSE | RPD | MAE | MBE | MAPE |
| --- | --- | --- | --- | --- | --- | --- | --- | --- | --- |
| traingdx | radbas-tansig | Training set | 0.89781 | 0.78006 | 0.60850 | 3.12920 | 0.60305 | 0.01898 | 0.05126 |
|  |  | Validation set | 0.81897 | 1.60020 | 2.56060 | 2.37510 | 1.01110 | 0.23059 | 0.19520 |
| traingdx | radbas-logsig | Training set | 0.49386 | 1.74500 | 3.04490 | 1.40560 | 1.20570 | 0.01157 | 0.19880 |
|  |  | Validation set | 0.67832 | 2.10910 | 4.44840 | 1.76320 | 1.32500 | 0.00736 | 0.15273 |
| traingdx | radbas-poslin | Training set | —— | | | | | | |
|  |  | Validation set |  |  |  |  |  |  |  |
| traingdx | radbas-purelin | Training set | 0.88196 | 0.99193 | 0.98392 | 2.91110 | 0.74302 | 0.01782 | 0.06323 |
|  |  | Validation set | 0.89448 | 0.95281 | 0.90784 | 3.08050 | 0.69553 | 0.03504 | 0.06094 |
| traingdx | logsig-tansig | Training set | 0.80537 | 1.36420 | 1.86100 | 2.26750 | 0.94288 | 0.03597 | 0.14486 |
|  |  | Validation set | 0.66735 | 1.33410 | 1.77980 | 1.80570 | 0.95473 | 0.37276 | 0.10997 |
| traingdx | logsig-logsig | Training set | 0.32475 | 2.44080 | 5.95740 | 1.52970 | 1.72120 | 1.47880 | 0.28646 |
|  |  | Validation set | 0.15602 | 2.51250 | 6.31260 | 1.22650 | 1.83120 | 1.15770 | 0.20921 |
| traingdx | logsig-poslin | Training set | —— | | | | | | |
|  |  | Validation set |  |  |  |  |  |  |  |
| traingdx | logsig-Purelin | Training set | —— | | | | | | |
|  |  | Validation set |  |  |  |  |  |  |  |
| traingdx | Tansing-tansig | Training set | 0.42935 | 2.24340 | 5.03270 | 1.35910 | 1.70720 | 0.50812 | 0.20906 |
|  |  | Validation set | 0.34994 | 2.18590 | 4.77810 | 1.55180 | 1.72240 | 1.31360 | 0.14181 |
| traingdx | Tansig-logsig | Training set | —— | | | | | | |
|  |  | Validation set |  |  |  |  |  |  |  |
| traingdx | Tansig-poslin | Training set | 0.57240 | 1.70120 | 2.89400 | 1.66730 | 1.13190 | 0.67772 | 0.13445 |
|  |  | Validation set | 0.57432 | 2.27210 | 5.16260 | 1.56060 | 1.39920 | 0.42746 | 0.34066 |
| traingdx | Tansig-Purelin | Training set | 0.30695 | 2.34120 | 5.48110 | 1.20190 | 1.75350 | 0.08011 | 0.20553 |
|  |  | Validation set | 0.15329 | 2.85530 | 8.15260 | 1.08700 | 1.93940 | 0.05638 | 0.15778 |
| traingdx | Purelin-tansig | Training set | 0.78224 | 0.85022 | 0.80292 | 2.81500 | 0.61255 | 0.07623 | 0.03947 |
|  |  | Validation set | 0.78741 | 0.96790 | 1.04050 | 2.88810 | 0.68501 | 0.02245 | 0.04421 |
| traingdx | Purelin-logsig | Training set | 0.43959 | 2.15780 | 4.65590 | 1.33790 | 1.55970 | 0.12147 | 0.23345 |
|  |  | Validation set | 0.69116 | 1.62850 | 2.65210 | 1.83270 | 1.11050 | 0.30889 | 0.09439 |
| traingdx | Purelin-poslin | Training set | 0.11228 | 2.45720 | 6.03770 | 1.06460 | 1.83430 | 0.19270 | 0.23610 |
|  |  | Validation set | 0.11060 | 3.26260 | 10.64470 | 1.06130 | 2.38620 | 0.13875 | 0.23384 |
| traingdx | Purelin-Purelin | Training set | 0.87299 | 0.99877 | 0.99755 | 2.80650 | 0.74850 | 0.02016 | 0.06653 |
|  |  | Validation set | 0.87398 | 1.10530 | 1.22160 | 2.81900 | 0.86277 | 0.04115 | 0.15380 |
| traingdx | poslin-tansig | Training set | —— | | | | | | |
|  |  | Validation set |  |  |  |  |  |  |  |
| traingdx | poslin-logsig | Training set | —— | | | | | | |
|  |  | Validation set |  |  |  |  |  |  |  |
| traingdx | poslin-poslin | Training set | 0.23424 | 2.36270 | 5.58210 | 1.17800 | 1.66550 | 0.57398 | 0.18125 |
|  |  | Validation set | 0.31922 | 2.70680 | 7.32690 | 1.22400 | 1.67720 | 0.37769 | 0.33682 |
| traingdx | poslin-Purelin | Training set | 0.81624 | 1.20210 | 1.44500 | 2.33910 | 0.93748 | 0.08817 | 0.08249 |
|  |  | Validation set | 0.59516 | 1.84240 | 3.39440 | 1.60560 | 1.10070 | 0.37686 | 0.34150 |
| Training function | Training function | Connection function | R2 | RMSE | MSE | RPD | MAE | MBE | MAPE |
| trainbr | radbas-tansig | Training set | 0.97645 | 0.46422 | 0.21550 | 6.51850 | 0.27241 | 0.01009 | 0.04798 |
|  |  | Validation set | 0.89011 | 0.84806 | 0.71920 | 3.06210 | 0.58376 | 0.14568 | 0.05134 |
| trainbr | radbas-logsig | Training set | —— | | | | | | |
|  |  | Validation set |  |  |  |  |  |  |  |
| trainbr | radbas-poslin | Training set | —— | | | | | | |
|  |  | Validation set |  |  |  |  |  |  |  |
| trainbr | radbas-purelin | Training set | 0.98270 | 0.41521 | 0.17240 | 7.60840 | 0.24036 | 0.01666 | 0.02119 |
|  |  | Validation set | 0.90839 | 0.66321 | 0.43984 | 3.45210 | 0.47601 | 0.19224 | 0.03936 |
| trainbr | logsig-tansig | Training set | 0.95997 | 0.57049 | 0.32546 | 5.00400 | 0.41430 | 0.02806 | 0.04837 |
|  |  | Validation set | 0.87730 | 1.05670 | 1.11650 | 2.87440 | 0.60528 | 0.12293 | 0.04969 |
| trainbr | logsig-logsig | Training set | 0.17915 | 2.62390 | 6.88500 | 1.31830 | 1.88310 | 1.43480 | 0.29567 |
|  |  | Validation set | 0.36256 | 2.31410 | 5.35490 | 1.45830 | 1.64520 | 1.18510 | 0.19923 |
| trainbr | logsig-poslin | Training set | —— | | | | | | |
|  |  | Validation set |  |  |  |  |  |  |  |
| trainbr | logsig-Purelin | Training set | 0.79969 | 1.21800 | 1.48360 | 2.23670 | 0.65935 | 0.05595 | 0.05161 |
|  |  | Validation set | 0.84658 | 1.28310 | 1.64640 | 2.61560 | 0.89772 | 0.27893 | 0.11993 |
| trainbr | Tansing-tansig | Training set | 0.96220 | 0.55603 | 0.30917 | 5.17470 | 0.31603 | 0.06111 | 0.02596 |
|  |  | Validation set | 0.94752 | 0.67801 | 0.45970 | 4.49300 | 0.42933 | 0.16056 | 0.10241 |
| trainbr | Tansig-logsig | Training set | 0.23147 | 2.73130 | 7.46000 | 1.46150 | 1.90890 | 1.70740 | 0.32396 |
|  |  | Validation set | 0.48676 | 1.57140 | 2.46930 | 1.63690 | 1.29270 | 0.82083 | 0.11590 |
| trainbr | Tansig-poslin | Training set | —— | | | | | | |
|  |  | Validation set |  |  |  |  |  |  |  |
| trainbr | Tansig-Purelin | Training set | 0.98109 | 0.34412 | 0.11842 | 7.28970 | 0.25379 | 0.02434 | 0.02189 |
|  |  | Validation set | 0.92964 | 0.94918 | 0.90094 | 3.85970 | 0.71211 | 0.20342 | 0.11015 |
| trainbr | Purelin-tansig | Training set | 0.82884 | 1.03140 | 1.06380 | 2.42010 | 0.59313 | 0.05153 | 0.05217 |
|  |  | Validation set | 0.77632 | 1.72410 | 2.97270 | 2.13820 | 1.07340 | 0.25680 | 0.14411 |
| trainbr | Purelin-logsig | Training set | 0.67722 | 1.63550 | 2.67500 | 1.82210 | 1.04360 | 0.42287 | 0.17965 |
|  |  | Validation set | 0.53630 | 2.00310 | 4.01230 | 1.56940 | 1.31740 | 0.70641 | 0.15969 |
| trainbr | Purelin-poslin | Training set | —— | | | | | | |
|  |  | Validation set |  |  |  |  |  |  |  |
| trainbr | Purelin-Purelin | Training set | 0.82194 | 1.19030 | 1.41670 | 2.38000 | 0.68349 | 0.10992 | 0.13600 |
|  |  | Validation set | 0.73378 | 1.58110 | 2.49980 | 1.95370 | 0.88012 | 0.19943 | 0.12904 |
| trainbr | poslin-tansig | Training set | 0.96108 | 0.56235 | 0.31624 | 5.07070 | 0.40087 | 0.01496 | 0.04949 |
|  |  | Validation set | 0.85523 | 1.13330 | 1.28450 | 2.69020 | 0.65294 | 0.24182 | 0.04666 |
| trainbr | poslin-logsig | Training set | 0.70440 | 1.60820 | 2.58640 | 1.91850 | 0.94909 | 0.45733 | 0.18595 |
|  |  | Validation set | 0.70457 | 1.50270 | 2.25810 | 1.88510 | 0.95933 | 0.32745 | 0.09281 |
| trainbr | poslin-poslin | Training set | —— | | | | | | |
|  |  | Validation set |  |  |  |  |  |  |  |
| trainbr | poslin-Purelin | Training set | 0.97526 | 0.46982 | 0.22073 | 6.35930 | 0.34213 | 0.01185 | 0.02814 |
|  |  | Validation set | 0.93341 | 0.66854 | 0.44694 | 3.87610 | 0.48163 | 0.01436 | 0.07483 |

| Training function | Training function | Connection function | R2 | RMSE | MSE | RPD | MAE | MBE | MAPE |
| --- | --- | --- | --- | --- | --- | --- | --- | --- | --- |
| trainlm | radbas-tansig | Training set | 0.94277 | 0.57555 | 0.33126 | 4.26230 | 0.42714 | 0.11260 | 0.03852 |
|  |  | Validation set | 0.76972 | 1.82990 | 3.34850 | 2.08420 | 1.02280 | 0.03133 | 0.20027 |
| trainlm | radbas-logsig | Training set | 0.25099 | 2.43500 | 5.92920 | 1.43960 | 1.72660 | 1.45240 | 0.27915 |
|  |  | Validation set | 0.37078 | 2.44660 | 5.98600 | 1.63740 | 1.73160 | 1.56140 | 0.21899 |
| trainlm | radbas-poslin | Training set | —— | | | | | | |
|  |  | Validation set |  |  |  |  |  |  |  |
| trainlm | radbas-purelin | Training set | 0.91527 | 0.82725 | 0.68434 | 3.60310 | 0.55612 | 0.24937 | 0.04367 |
|  |  | Validation set | 0.89514 | 0.97798 | 0.95645 | 3.10990 | 0.71880 | 0.11555 | 0.10896 |
| trainlm | logsig-tansig | Training set | 0.91705 | 0.75746 | 0.57374 | 3.47450 | 0.47808 | 0.02846 | 0.06235 |
|  |  | Validation set | 0.78363 | 1.55290 | 2.41160 | 2.19470 | 0.95080 | 0.31248 | 0.06484 |
| trainlm | logsig-logsig | Training set | 0.69943 | 1.61600 | 2.61150 | 1.89170 | 0.83201 | 0.42847 | 0.18443 |
|  |  | Validation set | 0.81471 | 1.17190 | 1.37320 | 2.37260 | 0.76151 | 0.23806 | 0.05984 |
| trainlm | logsig-poslin | Training set | 0.31137 | 2.63770 | 6.95750 | 1.43570 | 1.72680 | 1.43390 | 0.30474 |
|  |  | Validation set | 0.27231 | 1.80440 | 3.25590 | 1.66480 | 1.48180 | 1.28130 | 0.14267 |
| trainlm | logsig-Purelin | Training set | 0.95345 | 0.62490 | 0.39050 | 4.67600 | 0.40960 | 0.08247 | 0.03676 |
|  |  | Validation set | 0.93087 | 0.76485 | 0.58499 | 3.82770 | 0.59091 | 0.08615 | 0.04741 |
| trainlm | Tansing-tansig | Training set | 0.94820 | 0.69075 | 0.47713 | 4.47410 | 0.51816 | 0.13040 | 0.05456 |
|  |  | Validation set | 0.74840 | 1.28570 | 1.65300 | 2.00950 | 0.90648 | 0.16119 | 0.07343 |
| trainlm | Tansig-logsig | Training set | 0.56956 | 1.74360 | 3.04020 | 1.72520 | 1.03900 | 0.81678 | 0.13318 |
|  |  | Validation set | 0.60688 | 2.12790 | 4.52790 | 1.70480 | 1.17510 | 0.75171 | 0.31026 |
| trainlm | Tansig-poslin | Training set | 0.30129 | 2.48280 | 6.16430 | 1.49200 | 1.67880 | 1.48370 | 0.28906 |
|  |  | Validation set | 0.30148 | 2.28070 | 5.20170 | 1.38410 | 1.67090 | 1.14660 | 0.18443 |
| trainlm | Tansig-Purelin | Training set | 0.91868 | 0.90018 | 0.81033 | 3.51570 | 0.62215 | 0.06420 | 0.07667 |
|  |  | Validation set | 0.84712 | 0.84711 | 0.71760 | 2.73940 | 0.67038 | 0.30349 | 0.07060 |
| trainlm | Purelin-tansig | Training set | 0.94424 | 0.58290 | 0.33978 | 4.23470 | 0.42935 | 0.00170 | 0.03728 |
|  |  | Validation set | 0.91019 | 1.10060 | 1.21140 | 3.34530 | 0.63476 | 0.07796 | 0.11094 |
| trainlm | Purelin-logsig | Training set | —— | | | | | | |
|  |  | Validation set |  |  |  |  |  |  |  |
| trainlm | Purelin-poslin | Training set | 0.22586 | 2.63450 | 6.94080 | 1.44780 | 1.87160 | 1.63200 | 0.31270 |
|  |  | Validation set | 0.47240 | 1.86170 | 3.46610 | 1.59070 | 1.35280 | 0.93261 | 0.13741 |
| trainlm | Purelin-Purelin | Training set | 0.94485 | 0.69015 | 0.47631 | 4.26180 | 0.52712 | 0.02826 | 0.04299 |
|  |  | Validation set | 0.93680 | 0.69173 | 0.47850 | 3.98380 | 0.52480 | 0.03824 | 0.08603 |
| trainlm | poslin-tansig | Training set | 0.95172 | 0.56121 | 0.31495 | 4.64010 | 0.41651 | 0.10927 | 0.03673 |
|  |  | Validation set | 0.87950 | 1.23370 | 1.52210 | 2.90000 | 0.72628 | 0.14187 | 0.12118 |
| trainlm | poslin-logsig | Training set | 0.67962 | 1.58540 | 2.51340 | 1.85110 | 0.72166 | 0.47324 | 0.17408 |
|  |  | Validation set | 0.80820 | 1.33210 | 1.77440 | 2.31360 | 0.90286 | 0.21447 | 0.07282 |
| trainlm | poslin-poslin | Training set | 0.19606 | 2.71840 | 7.38940 | 1.39790 | 1.89200 | 1.63890 | 0.32089 |
|  |  | Validation set | 0.56371 | 1.59640 | 2.54850 | 1.80000 | 1.29620 | 0.86353 | 0.11550 |
| trainlm | poslin-Purelin | Training set | 0.95894 | 0.36339 | 0.12699 | 6.91110 | 0.23466 | 0.02167 | 0.00092 |
|  |  | Validation set | 0.93874 | 0.67060 | 0.45693 | 4.94670 | 0.48930 | 0.07133 | 0.04149 |

**Note:** The dashes indicate invalid entries.

**TABLE 3:** Model indicators for titratable acid of Gray Jujube based on ANN algorithm.

| Training function | Training function | Connection function | R2 | RMSE | MSE | RPD | MAE | MBE | MAPE |
| --- | --- | --- | --- | --- | --- | --- | --- | --- | --- |
| traingdx | radbas-tansig | Training set | 0.81329 | 0.03229 | 0.00104 | 2.37820 | 0.02066 | 0.00744 | 0.04287 |
|  |  | Validation set | 0.59239 | 0.05542 | 0.00307 | 1.58330 | 0.04041 | 0.00810 | 0.09757 |
| traingdx | radbas-logsig | Training set | 0.61038 | 0.04951 | 0.00245 | 1.65700 | 0.03046 | 0.01265 | 0.07634 |
|  |  | Validation set | 0.57085 | 0.05034 | 0.00253 | 1.53530 | 0.03278 | 0.00538 | 0.07207 |
| traingdx | radbas-poslin | Training set | 0.28703 | 0.06155 | 0.00379 | 1.31870 | 0.04672 | 0.02707 | 0.10525 |
|  |  | Validation set | 0.06475 | 0.08749 | 0.00765 | 1.06740 | 0.05879 | 0.02170 | 0.14425 |
| traingdx | radbas-purelin | Training set | 0.73876 | 0.07769 | 0.10896 | 2.46390 | 0.08797 | 0.10858 | 0.06042 |
|  |  | Validation set | 0.66885 | 0.07860 | 0.10901 | 2.13940 | 0.08478 | 0.09976 | 0.05734 |
| traingdx | logsig-tansig | Training set | 0.74338 | 0.04112 | 0.00169 | 2.00030 | 0.02920 | 0.00664 | 0.06376 |
|  |  | Validation set | 0.66471 | 0.04153 | 0.00173 | 1.78020 | 0.03412 | 0.01008 | 0.07074 |
| traingdx | logsig-logsig | Training set | 0.29240 | 0.06757 | 0.00457 | 1.32410 | 0.04878 | 0.02976 | 0.12111 |
|  |  | Validation set | 0.38186 | 0.05824 | 0.00339 | 1.35370 | 0.04546 | 0.01994 | 0.10093 |
| traingdx | logsig-poslin | Training set | 0.11338 | 0.07709 | 0.00594 | 1.07540 | 0.05791 | 0.01214 | 0.00594 |
|  |  | Validation set | 0.00011 | 0.06971 | 0.00486 | 1.06110 | 0.05151 | 0.02331 | 0.12489 |
| traingdx | logsig-Purelin | Training set | 0.32005 | 0.06055 | 0.00367 | 1.21850 | 0.05018 | 0.00586 | 0.11160 |
|  |  | Validation set | 0.34307 | 0.07049 | 0.00497 | 1.23390 | 0.05725 | 0.00077 | 0.11632 |
| traingdx | Tansing-tansig | Training set | 0.69524 | 0.04437 | 0.00197 | 1.81590 | 0.03447 | 0.00312 | 0.07391 |
|  |  | Validation set | 0.64934 | 0.04394 | 0.00193 | 1.69000 | 0.02908 | 0.00172 | 0.07134 |
| traingdx | Tansig-logsig | Training set | 0.37007 | 0.06055 | 0.00367 | 1.48300 | 0.04710 | 0.03193 | 0.11033 |
|  |  | Validation set | 0.29811 | 0.07018 | 0.00493 | 1.37150 | 0.04759 | 0.03456 | 0.12392 |
| traingdx | Tansig-poslin | Training set | 0.25683 | 0.07066 | 0.00499 | 1.19950 | 0.05311 | 0.01799 | 0.12490 |
|  |  | Validation set | 0.16167 | 0.06411 | 0.00411 | 1.11500 | 0.05191 | 0.01291 | 0.11695 |
| traingdx | Tansig-Purelin | Training set | 0.67085 | 0.04687 | 0.00220 | 1.74310 | 0.03744 | 0.00021 | 0.07774 |
|  |  | Validation set | 0.47537 | 0.04771 | 0.00228 | 1.60070 | 0.03727 | 0.02414 | 0.08900 |
| traingdx | Purelin-tansig | Training set | 0.74018 | 0.03653 | 0.00133 | 1.96200 | 0.02827 | 0.00045 | 0.05875 |
|  |  | Validation set | 0.69630 | 0.05088 | 0.00259 | 1.81530 | 0.03639 | 0.00140 | 0.08326 |
| traingdx | Purelin-logsig | Training set | 0.42091 | 0.06158 | 0.00379 | 1.53260 | 0.04182 | 0.03169 | 0.10622 |
|  |  | Validation set | 0.41409 | 0.05572 | 0.00310 | 1.46130 | 0.04266 | 0.02497 | 0.09824 |
| traingdx | Purelin-poslin | Training set | 0.15490 | 0.07476 | 0.00559 | 1.10590 | 0.05638 | 0.01347 | 0.13391 |
|  |  | Validation set | 0.09747 | 0.06815 | 0.00464 | 1.05940 | 0.04942 | 0.00770 | 0.10324 |
| traingdx | Purelin-Purelin | Training set | 0.73925 | 0.08970 | 0.11908 | 2.54990 | 0.09925 | 0.11826 | 0.07626 |
|  |  | Validation set | 0.69033 | 0.08920 | 0.11905 | 2.18270 | 0.09767 | 0.11768 | 0.07216 |
| traingdx | poslin-tansig | Training set | 0.72355 | 0.01498 | 0.04877 | 2.05180 | 0.02544 | 0.04933 | 0.00135 |
|  |  | Validation set | 0.73024 | 0.00918 | 0.04833 | 2.08430 | 0.02121 | 0.04865 | 0.01623 |
| traingdx | poslin-logsig | Training set | 0.24065 | 0.06744 | 0.00455 | 1.17160 | 0.04870 | 0.01360 | 0.10748 |
|  |  | Validation set | 0.40449 | 0.06266 | 0.00393 | 1.29650 | 0.04727 | 0.00203 | 0.10609 |
| traingdx | poslin-poslin | Training set | —— | | | | | | |
|  |  | Validation set |  |  |  |  |  |  |  |
| traingdx | poslin-Purelin | Training set | 0.74553 | 0.01325 | 0.04865 | 2.16240 | 0.02345 | 0.04893 | 0.00344 |
|  |  | Validation set | 0.65703 | 0.01114 | 0.04849 | 1.86170 | 0.02060 | 0.04001 | 0.01725 |
| Training function | Training function | Connection function | R2 | RMSE | MSE | RPD | MAE | MBE | MAPE |
| trainbr | radbas-tansig | Training set | 0.88735 | 0.02796 | 0.00078 | 2.85620 | 0.02083 | 0.00066 | 0.04307 |
|  |  | Validation set | 0.86619 | 0.02722 | 0.00074 | 2.83060 | 0.02030 | 0.00706 | 0.04176 |
| trainbr | radbas-logsig | Training set | 0.37363 | 0.05444 | 0.00296 | 1.50030 | 0.03967 | 0.02935 | 0.09360 |
|  |  | Validation set | 0.32998 | 0.08009 | 0.00641 | 1.36130 | 0.06068 | 0.03533 | 0.15324 |
| trainbr | radbas-poslin | Training set | —— | | | | | | |
|  |  | Validation set |  |  |  |  |  |  |  |
| trainbr | radbas-purelin | Training set | —— | | | | | | |
|  |  | Validation set |  |  |  |  |  |  |  |
| trainbr | logsig-tansig | Training set | 0.86556 | 0.02965 | 0.00088 | 2.72900 | 0.02157 | 0.00105 | 0.04542 |
|  |  | Validation set | 0.86000 | 0.02679 | 0.00072 | 2.82890 | 0.02314 | 0.00878 | 0.04943 |
| trainbr | logsig-logsig | Training set | 0.42846 | 0.05843 | 0.00341 | 1.48760 | 0.04001 | 0.02674 | 0.09913 |
|  |  | Validation set | 0.31328 | 0.06719 | 0.00451 | 1.32750 | 0.04852 | 0.02800 | 0.11748 |
| trainbr | logsig-poslin | Training set | 0.62962 | 0.04732 | 0.00224 | 1.70070 | 0.02678 | 0.01221 | 0.06918 |
|  |  | Validation set | 0.71896 | 0.04236 | 0.00179 | 1.88660 | 0.02886 | 0.07550 | 0.05825 |
| trainbr | logsig-Purelin | Training set | 0.86911 | 0.02777 | 0.00077 | 2.76870 | 0.01911 | 0.00160 | 0.04128 |
|  |  | Validation set | 0.88498 | 0.02798 | 0.00078 | 2.96110 | 0.02120 | 0.00257 | 0.04302 |
| trainbr | Tansing-tansig | Training set | 0.92500 | 0.02034 | 0.00041 | 3.65660 | 0.01150 | 0.00108 | 0.02316 |
|  |  | Validation set | 0.68209 | 0.04953 | 0.00245 | 1.80680 | 0.03103 | 0.00945 | 0.05845 |
| trainbr | Tansig-logsig | Training set | 0.47195 | 0.06106 | 0.00373 | 1.58210 | 0.04365 | 0.03013 | 0.10802 |
|  |  | Validation set | 0.25170 | 0.05946 | 0.00354 | 1.64970 | 0.04645 | 0.04242 | 0.11274 |
| trainbr | Tansig-poslin | Training set | 0.72336 | 0.03862 | 0.00149 | 2.02390 | 0.01694 | 0.01324 | 0.04956 |
|  |  | Validation set | 0.87637 | 0.03058 | 0.00094 | 2.92500 | 0.01720 | 0.00714 | 0.03931 |
| trainbr | Tansig-Purelin | Training set | 0.83889 | 0.02682 | 0.00072 | 2.49200 | 0.01869 | 0.00062 | 0.03818 |
|  |  | Validation set | 0.85920 | 0.03502 | 0.00123 | 2.66980 | 0.02414 | 0.00209 | 0.05306 |
| trainbr | Purelin-tansig | Training set | 0.87151 | 0.02940 | 0.00086 | 2.79360 | 0.01992 | 0.00156 | 0.04209 |
|  |  | Validation set | 0.80309 | 0.03094 | 0.00096 | 2.33020 | 0.02070 | 0.00787 | 0.04143 |
| trainbr | Purelin-logsig | Training set | —— | | | | | | |
|  |  | Validation set |  |  |  |  |  |  |  |
| trainbr | Purelin-poslin | Training set | 0.67227 | 0.04491 | 0.00202 | 1.79920 | 0.02656 | 0.01076 | 0.06536 |
|  |  | Validation set | 0.63622 | 0.04763 | 0.00227 | 1.67860 | 0.03111 | 0.00744 | 0.07111 |
| trainbr | Purelin-Purelin | Training set | 0.87716 | 0.02828 | 0.00080 | 2.85310 | 0.01996 | 0.00016 | 0.04208 |
|  |  | Validation set | 0.84885 | 0.02851 | 0.00081 | 2.57430 | 0.02174 | 0.00158 | 0.04400 |
| trainbr | poslin-tansig | Training set | 0.90109 | 0.02404 | 0.00058 | 3.17990 | 0.01531 | 0.00026 | 0.03222 |
|  |  | Validation set | 0.78501 | 0.03862 | 0.00149 | 2.33320 | 0.02739 | 0.01473 | 0.06392 |
| trainbr | poslin-logsig | Training set | 0.32479 | 0.06501 | 0.00423 | 1.44530 | 0.04415 | 0.03507 | 0.11524 |
|  |  | Validation set | 0.57284 | 0.04879 | 0.00238 | 1.59210 | 0.03879 | 0.01349 | 0.07981 |
| trainbr | poslin-poslin | Training set | 0.43352 | 0.05599 | 0.00314 | 1.47920 | 0.03863 | 0.02461 | 0.09247 |
|  |  | Validation set | 0.44547 | 0.06495 | 0.00422 | 1.58100 | 0.04765 | 0.03428 | 0.12163 |
| trainbr | poslin-Purelin | Training set | 0.87811 | 0.02646 | 0.00070 | 2.86640 | 0.02101 | 0.00102 | 0.04315 |
|  |  | Validation set | 0.83775 | 0.03388 | 0.00115 | 2.57450 | 0.02615 | 0.00897 | 0.05665 |

| Training function | Training function | Connection function | R2 | RMSE | MSE | RPD | MAE | MBE | MAPE |
| --- | --- | --- | --- | --- | --- | --- | --- | --- | --- |
| trainlm | radbas-tansig | Training set | 0.82992 | 0.03392 | 0.00115 | 2.42720 | 0.02412 | 0.00151 | 0.05214 |
|  |  | Validation set | 0.76265 | 0.03271 | 0.00107 | 2.14070 | 0.02487 | 0.00929 | 0.05475 |
| trainlm | radbas-logsig | Training set | 0.24900 | 0.07243 | 0.00525 | 1.26580 | 0.04857 | 0.02977 | 0.12157 |
|  |  | Validation set | 0.22240 | 0.05710 | 0.00326 | 1.35440 | 0.04417 | 0.03122 | 0.10273 |
| trainlm | radbas-poslin | Training set | 0.45219 | 0.06053 | 0.00366 | 1.48270 | 0.03588 | 0.24929 | 0.09291 |
|  |  | Validation set | 0.07089 | 0.06717 | 0.00451 | 1.12050 | 0.05099 | 0.02538 | 0.11787 |
| trainlm | radbas-purelin | Training set | 0.85467 | 0.03061 | 0.00094 | 2.62430 | 0.02193 | 0.00091 | 0.04479 |
|  |  | Validation set | 0.47840 | 0.05377 | 0.00289 | 1.39880 | 0.03427 | 0.00764 | 0.07362 |
| trainlm | logsig-tansig | Training set | 0.92601 | 0.02211 | 0.00049 | 3.67790 | 0.01530 | 0.00067 | 0.03121 |
|  |  | Validation set | 0.85376 | 0.02748 | 0.00076 | 2.65060 | 0.01991 | 0.00449 | 0.04067 |
| trainlm | logsig-logsig | Training set | 0.67774 | 0.04256 | 0.00181 | 1.78090 | 0.02550 | 0.00626 | 0.06293 |
|  |  | Validation set | 0.69769 | 0.04705 | 0.00221 | 1.89040 | 0.03115 | 0.01283 | 0.07485 |
| trainlm | logsig-poslin | Training set | 0.43812 | 0.06078 | 0.00369 | 1.50730 | 0.03879 | 0.02829 | 0.10014 |
|  |  | Validation set | 0.28814 | 0.06111 | 0.00373 | 1.31290 | 0.04536 | 0.02629 | 0.10370 |
| trainlm | logsig-Purelin | Training set | 0.93539 | 0.01873 | 0.00035 | 3.93570 | 0.01358 | 0.00053 | 0.02963 |
|  |  | Validation set | 0.26854 | 0.07406 | 0.00549 | 1.22400 | 0.04362 | 0.02161 | 0.07660 |
| trainlm | Tansing-tansig | Training set | 0.86245 | 0.02801 | 0.00078 | 2.75460 | 0.02092 | 0.00573 | 0.04345 |
|  |  | Validation set | 0.76624 | 0.04127 | 0.00170 | 2.12490 | 0.03002 | 0.00946 | 0.07151 |
| trainlm | Tansig-logsig | Training set | 0.38698 | 0.05338 | 0.00285 | 1.45190 | 0.03428 | 0.02539 | 0.08502 |
|  |  | Validation set | 0.46418 | 0.07094 | 0.00503 | 1.38650 | 0.05016 | 0.01213 | 0.12307 |
| trainlm | Tansig-poslin | Training set | 0.38252 | 0.06361 | 0.00405 | 1.38580 | 0.04364 | 0.02518 | 0.10922 |
|  |  | Validation set | 0.37585 | 0.05736 | 0.00329 | 1.33980 | 0.04397 | 0.01880 | 0.09857 |
| trainlm | Tansig-Purelin | Training set | 0.89548 | 0.02555 | 0.00065 | 3.09410 | 0.01519 | 0.00064 | 0.03267 |
|  |  | Validation set | 0.70480 | 0.04212 | 0.00177 | 1.84060 | 0.03322 | 0.00049 | 0.06878 |
| trainlm | Purelin-tansig | Training set | 0.88434 | 0.02935 | 0.00086 | 2.94240 | 0.01845 | 0.00107 | 0.03874 |
|  |  | Validation set | 0.77436 | 0.02682 | 0.00072 | 2.15410 | 0.01956 | 0.00568 | 0.04022 |
| trainlm | Purelin-logsig | Training set | 0.32682 | 0.06370 | 0.00406 | 1.28710 | 0.04497 | 0.02047 | 0.10899 |
|  |  | Validation set | 0.22735 | 0.07100 | 0.00504 | 1.21460 | 0.05608 | 0.02486 | 0.12736 |
| trainlm | Purelin-poslin | Training set | 0.00550 | 0.07719 | 0.00596 | 1.01130 | 0.05692 | 0.01003 | 0.13450 |
|  |  | Validation set | 0.13908 | 0.07352 | 0.00541 | 1.08140 | 0.05404 | 0.00606 | 0.10928 |
| trainlm | Purelin-Purelin | Training set | 0.84404 | 0.02984 | 0.00089 | 2.53940 | 0.01938 | 0.00225 | 0.04258 |
|  |  | Validation set | 0.89528 | 0.02716 | 0.00074 | 3.11060 | 0.01936 | 0.00311 | 0.04112 |
| trainlm | poslin-tansig | Training set | 0.72739 | 0.03911 | 0.00153 | 2.00500 | 0.02892 | 0.01157 | 0.06790 |
|  |  | Validation set | 0.73422 | 0.04435 | 0.00197 | 2.13090 | 0.03640 | 0.01836 | 0.07811 |
| trainlm | poslin-logsig | Training set | 0.65599 | 0.04570 | 0.00209 | 1.76420 | 0.02563 | 0.01174 | 0.06692 |
|  |  | Validation set | 0.35820 | 0.06388 | 0.00408 | 1.27480 | 0.04551 | 0.01298 | 0.09269 |
| trainlm | poslin-poslin | Training set | 0.69428 | 0.04379 | 0.00192 | 1.89730 | 0.02559 | 0.01324 | 0.06533 |
|  |  | Validation set | 0.75496 | 0.03821 | 0.00146 | 2.13500 | 0.02593 | 0.01237 | 0.06125 |
| trainlm | poslin-Purelin | Training set | 0.90344 | 0.02445 | 0.00060 | 3.23290 | 0.01582 | 0.00234 | 0.03214 |
|  |  | Validation set | 0.74962 | 0.03915 | 0.00153 | 2.00150 | 0.02788 | 0.00213 | 0.06242 |

**Note:** The dashes indicate invalid entries.

**TABLE 4:** Grey jujube sugar-acid ratio model indicators based on ANN algorithm.

| Training function | Training function | Connection function | R2 | RMSE | MSE | RPD | MAE | MBE | MAPE |
| --- | --- | --- | --- | --- | --- | --- | --- | --- | --- |
| traingdx | radbas-tansig | Training set | 0.02172 | 7.07740 | 50.08990 | 1.01530 | 5.40010 | 0.64773 | 0.23057 |
|  |  | Validation set | 0.14462 | 8.09820 | 65.58080 | 1.08140 | 6.37870 | 0.13101 | 0.34342 |
| traingdx | radbas-logsig | Training set | 0.13948 | 6.81070 | 46.38600 | 1.52030 | 5.27780 | 4.80260 | 0.28870 |
|  |  | Validation set | 0.17680 | 7.61160 | 57.93690 | 1.41710 | 5.48150 | 4.78450 | 0.46129 |
| traingdx | radbas-poslin | Training set | —— | | | | | | |
|  |  | Validation set |  |  |  |  |  |  |  |
| traingdx | radbas-purelin | Training set | 0.92108 | 2.20930 | 4.88700 | 3.56420 | 1.70590 | 0.11202 | 0.08618 |
|  |  | Validation set | 0.84362 | 2.81640 | 7.93200 | 2.52980 | 2.28630 | 0.08064 | 0.09415 |
| traingdx | logsig-tansig | Training set | 0.27180 | 6.42060 | 41.22410 | 1.31810 | 5.18220 | 2.93930 | 0.31489 |
|  |  | Validation set | 0.36476 | 6.38020 | 40.70650 | 1.34570 | 5.37910 | 2.30660 | 0.26208 |
| traingdx | logsig-logsig | Training set | —— | | | | | | |
|  |  | Validation set |  |  |  |  |  |  |  |
| traingdx | logsig-poslin | Training set | —— | | | | | | |
|  |  | Validation set |  |  |  |  |  |  |  |
| traingdx | logsig-Purelin | Training set | 0.43016 | 5.56160 | 3.09319 | 1.34840 | 4.58890 | 1.03840 | 0.21112 |
|  |  | Validation set | 0.36421 | 6.64990 | 44.22170 | 1.25470 | 5.38600 | 0.19120 | 0.24550 |
| traingdx | Tansing-tansig | Training set | 0.90132 | 2.28570 | 5.22450 | 3.18350 | 1.72830 | 0.00854 | 0.09390 |
|  |  | Validation set | 0.86700 | 3.08110 | 9.49330 | 2.74650 | 2.32590 | 0.17568 | 0.10865 |
| traingdx | Tansig-logsig | Training set | 0.45502 | 5.88460 | 34.62860 | 1.56980 | 4.41380 | 2.97410 | 0.30032 |
|  |  | Validation set | 0.38955 | 5.38510 | 28.99900 | 1.54740 | 4.38620 | 3.02650 | 0.21888 |
| traingdx | Tansig-poslin | Training set | —— | | | | | | |
|  |  | Validation set |  |  |  |  |  |  |  |
| traingdx | Tansig-Purelin | Training set | 0.37954 | 6.19120 | 38.33080 | 1.31330 | 5.36350 | 1.58540 | 0.24373 |
|  |  | Validation set | 0.25670 | 6.06410 | 36.77370 | 1.18690 | 4.76810 | 1.28710 | 0.19912 |
| traingdx | Purelin-tansig | Training set | —— | | | | | | |
|  |  | Validation set |  |  |  |  |  |  |  |
| traingdx | Purelin-logsig | Training set | 0.26168 | 6.85530 | 46.99450 | 1.19540 | 5.37140 | 1.56610 | 0.30979 |
|  |  | Validation set | 0.10105 | 6.37250 | 40.60820 | 1.14720 | 5.08430 | 2.50820 | 0.26640 |
| traingdx | Purelin-poslin | Training set | 0.17319 | 6.75230 | 45.59380 | 1.14110 | 4.94540 | 1.80090 | 0.30511 |
|  |  | Validation set | 0.46443 | 5.92820 | 35.14300 | 1.43930 | 4.48200 | 1.86220 | 0.21145 |
| traingdx | Purelin-Purelin | Training set | 0.92211 | 2.08570 | 4.35000 | 3.58320 | 1.65680 | 0.00828 | 0.07030 |
|  |  | Validation set | 0.87791 | 2.82830 | 7.99920 | 2.86400 | 2.21690 | 0.10833 | 0.09779 |
| traingdx | poslin-tansig | Training set | 0.72771 | 3.96880 | 15.75130 | 1.92480 | 2.85570 | 0.37011 | 0.12064 |
|  |  | Validation set | 0.62329 | 4.76870 | 22.74030 | 1.67570 | 3.52710 | 1.11460 | 0.21165 |
| traingdx | poslin-logsig | Training set | —— | | | | | | |
|  |  | Validation set |  |  |  |  |  |  |  |
| traingdx | poslin-poslin | Training set | 0.49595 | 4.97270 | 24.72750 | 1.46760 | 3.62150 | 1.39650 | 0.23667 |
|  |  | Validation set | 0.54840 | 6.06130 | 36.73940 | 1.50760 | 4.89910 | 0.97295 | 0.20101 |
| traingdx | poslin-Purelin | Training set | 0.49020 | 5.58760 | 31.22150 | 1.40090 | 3.91550 | 0.12865 | 0.20527 |
|  |  | Validation set | 0.55152 | 4.85180 | 23.54000 | 1.50260 | 3.57340 | 0.54196 | 0.14527 |
| Training function | Training function | Connection function | R2 | RMSE | MSE | RPD | MAE | MBE | MAPE |
| trainbr | radbas-tansig | Training set | 0.97231 | 1.33270 | 1.77610 | 6.07480 | 0.60513 | 0.19511 | 0.02641 |
|  |  | Validation set | 0.66798 | 3.91190 | 15.30310 | 1.83850 | 1.79970 | 0.12909 | 0.08527 |
| trainbr | radbas-logsig | Training set | 0.51046 | 5.34610 | 28.58050 | 1.70370 | 3.48590 | 2.90950 | 0.25841 |
|  |  | Validation set | 0.57804 | 5.02540 | 25.25420 | 1.81390 | 3.35790 | 2.65780 | 0.19388 |
| trainbr | radbas-poslin | Training set | —— | | | | | | |
|  |  | Validation set |  |  |  |  |  |  |  |
| trainbr | radbas-purelin | Training set | 0.94801 | 0.78696 | 0.64386 | 9.20310 | 0.32682 | 0.08646 | 0.02574 |
|  |  | Validation set | 0.88017 | 2.18370 | 4.90500 | 3.50520 | 1.12520 | 0.08838 | 0.07357 |
| trainbr | logsig-tansig | Training set | 0.93943 | 1.93070 | 3.72750 | 4.06860 | 0.92277 | 0.28889 | 0.01579 |
|  |  | Validation set | 0.63407 | 4.30810 | 18.56010 | 1.68290 | 1.92440 | 2.11877 | 0.08771 |
| trainbr | logsig-logsig | Training set | 0.55681 | 5.11990 | 26.21360 | 1.79860 | 26.21360 | 5.11990 | 0.24784 |
|  |  | Validation set | 0.51486 | 5.29550 | 28.04230 | 1.78920 | 3.62400 | 3.16000 | 0.20881 |
| trainbr | logsig-poslin | Training set | —— | | | | | | |
|  |  | Validation set |  |  |  |  |  |  |  |
| trainbr | logsig-Purelin | Training set | 0.98873 | 0.84772 | 0.71863 | 9.44560 | 0.51923 | 0.06473 | 0.02269 |
|  |  | Validation set | 0.80607 | 3.01200 | 9.07220 | 2.57480 | 2.07910 | 1.41980 | 0.10039 |
| trainbr | Tansing-tansig | Training set | 0.93903 | 1.81710 | 3.30190 | 4.05530 | 0.66828 | 0.95233 | 0.02719 |
|  |  | Validation set | 0.86886 | 2.98690 | 8.92170 | 2.87230 | 1.94790 | 0.82180 | 0.14119 |
| trainbr | Tansig-logsig | Training set | 0.52854 | 5.28690 | 27.95110 | 1.78290 | 3.49820 | 3.04950 | 0.26078 |
|  |  | Validation set | 0.55747 | 5.05710 | 25.57460 | 1.95280 | 3.66840 | 3.22810 | 0.20450 |
| trainbr | Tansig-poslin | Training set | —— | | | | | | |
|  |  | Validation set |  |  |  |  |  |  |  |
| trainbr | Tansig-Purelin | Training set | 0.98275 | 0.94627 | 0.89543 | 7.63400 | 0.06753 | 0.89543 | 0.02997 |
|  |  | Validation set | 0.86231 | 3.14440 | 9.88720 | 2.72730 | 2.52290 | 0.48305 | 3.14440 |
| trainbr | Purelin-tansig | Training set | 0.94922 | 1.35270 | 1.86420 | 5.68090 | 1.04680 | 0.01799 | 0.03069 |
|  |  | Validation set | 0.93687 | 1.49540 | 2.27650 | 5.12540 | 1.16740 | 0.51411 | 0.03203 |
| trainbr | Purelin-logsig | Training set | 0.51406 | 5.46700 | 29.88830 | 1.80680 | 3.56150 | 3.32390 | 0.26690 |
|  |  | Validation set | 0.54583 | 4.88600 | 23.87290 | 1.86900 | 3.26210 | 2.97080 | 0.19158 |
| trainbr | Purelin-poslin | Training set | —— | | | | | | |
|  |  | Validation set |  |  |  |  |  |  |  |
| trainbr | Purelin-Purelin | Training set | 0.95425 | 1.73290 | 3.00300 | 4.67700 | 1.28200 | 0.04609 | 0.05990 |
|  |  | Validation set | 0.90798 | 1.98470 | 3.93910 | 3.30840 | 1.51920 | 0.16785 | 0.06292 |
| trainbr | poslin-tansig | Training set | 0.93135 | 1.59150 | 2.57690 | 4.52380 | 1.11420 | 0.08774 | 0.03557 |
|  |  | Validation set | 0.91267 | 2.13420 | 4.62060 | 3.91810 | 1.54780 | 0.42368 | 0.04672 |
| trainbr | poslin-logsig | Training set | 0.39998 | 5.63130 | 31.71170 | 1.57790 | 3.91450 | 3.23780 | 0.28071 |
|  |  | Validation set | 0.61627 | 5.19160 | 26.95240 | 1.91720 | 3.94670 | 2.80080 | 0.20955 |
| trainbr | poslin-poslin | Training set | 0.50999 | 5.71670 | 32.68090 | 1.71620 | 3.93070 | 3.16800 | 0.28821 |
|  |  | Validation set | 0.48908 | 4.54330 | 20.64130 | 1.71000 | 3.24200 | 2.61240 | 0.16864 |
| trainbr | poslin-Purelin | Training set | 0.96685 | 1.44530 | 2.08890 | 5.49300 | 1.66300 | 0.02200 | 0.04631 |
|  |  | Validation set | 0.89139 | 2.29670 | 5.27500 | 3.08010 | 1.70850 | 0.39415 | 0.08208 |

| Training function | Training function | Connection function | R2 | RMSE | MSE | RPD | MAE | MBE | MAPE |
| --- | --- | --- | --- | --- | --- | --- | --- | --- | --- |
| trainlm | radbas-tansig | Training set | 0.84417 | 3.11320 | 9.69180 | 2.53890 | 1.96130 | 0.20688 | 0.14994 |
|  |  | Validation set | 0.84798 | 2.72590 | 7.43050 | 2.56500 | 1.93610 | 0.03296 | 0.09826 |
| trainlm | radbas-logsig | Training set | 0.42605 | 5.68970 | 32.37210 | 1.53380 | 3.73590 | 2.89780 | 0.27620 |
|  |  | Validation set | 0.62560 | 4.87190 | 23.73510 | 1.83330 | 3.49440 | 2.20770 | 0.18103 |
| trainlm | radbas-poslin | Training set | —— | | | | | | |
|  |  | Validation set |  |  |  |  |  |  |  |
| trainlm | radbas-purelin | Training set | 0.89195 | 2.56310 | 6.56940 | 3.09940 | 1.85170 | 0.49012 | 0.08441 |
|  |  | Validation set | 0.86972 | 2.65500 | 7.04920 | 3.01680 | 2.23160 | 1.05070 | 0.10608 |
| trainlm | logsig-tansig | Training set | 0.92936 | 1.82550 | 3.33250 | 3.76390 | 1.30920 | 0.05064 | 0.06926 |
|  |  | Validation set | 0.95156 | 2.02430 | 4.09760 | 4.58540 | 1.59030 | 0.27320 | 0.06804 |
| trainlm | logsig-logsig | Training set | 0.56018 | 5.31100 | 28.20630 | 1.81650 | 3.32780 | 2.96140 | 0.25525 |
|  |  | Validation set | 0.40687 | 5.21610 | 27.20770 | 1.61280 | 3.76220 | 3.09400 | 0.21548 |
| trainlm | logsig-poslin | Training set | 0.60140 | 4.54380 | 20.64600 | 1.77640 | 2.77100 | 2.05720 | 0.21448 |
|  |  | Validation set | 0.67308 | 4.96270 | 24.62800 | 2.01610 | 3.37920 | 2.46880 | 0.20678 |
| trainlm | logsig-Purelin | Training set | 0.73871 | 4.00770 | 16.06200 | 1.97850 | 2.93130 | 0.59867 | 0.17837 |
|  |  | Validation set | 0.72123 | 3.79270 | 14.38440 | 1.89460 | 3.21530 | 0.09886 | 0.13622 |
| trainlm | Tansing-tansig | Training set | 0.93756 | 1.89870 | 3.60510 | 4.04720 | 1.51750 | 0.28305 | 0.07767 |
|  |  | Validation set | 0.91745 | 2.23680 | 5.00330 | 3.49040 | 1.64980 | 0.16766 | 0.07443 |
| trainlm | Tansig-logsig | Training set | 0.53267 | 5.59960 | 31.35580 | 1.79380 | 3.42550 | 3.24080 | 0.27232 |
|  |  | Validation set | 0.46552 | 4.59030 | 21.07090 | 1.77660 | 3.43950 | 2.92930 | 0.17119 |
| trainlm | Tansig-poslin | Training set | 0.46356 | 5.75920 | 33.16830 | 1.64440 | 3.20980 | 3.31683 | 0.28413 |
|  |  | Validation set | 0.69765 | 3.90590 | 15.25600 | 2.32000 | 2.84720 | 3.24251 | 0.13869 |
| trainlm | Tansig-Purelin | Training set | 0.94960 | 1.75390 | 3.07630 | 4.46670 | 1.23260 | 0.12984 | 0.05516 |
|  |  | Validation set | 0.88557 | 2.47850 | 6.14280 | 3.12080 | 1.87510 | 0.79424 | 0.08898 |
| trainlm | Purelin-tansig | Training set | 0.93125 | 1.89980 | 3.60920 | 3.88010 | 1.48860 | 0.34947 | 0.07340 |
|  |  | Validation set | 0.92554 | 2.33650 | 5.45930 | 3.73710 | 1.60230 | 0.45750 | 0.07556 |
| trainlm | Purelin-logsig | Training set | 0.49852 | 5.80720 | 33.72330 | 1.69060 | 3.78980 | 3.19290 | 0.28744 |
|  |  | Validation set | 0.49300 | 4.45250 | 19.82510 | 1.56750 | 3.26720 | 1.97760 | 0.15288 |
| trainlm | Purelin-poslin | Training set | 0.53655 | 4.82610 | 23.29130 | 1.76010 | 3.00240 | 2.65870 | 0.22884 |
|  |  | Validation set | 0.50159 | 6.26890 | 39.29950 | 1.73870 | 4.49510 | 3.63530 | 0.27393 |
| trainlm | Purelin-Purelin | Training set | 0.94565 | 1.78950 | 3.20220 | 4.28960 | 1.31520 | 0.01077 | 0.05890 |
|  |  | Validation set | 0.91980 | 2.16810 | 4.70050 | 3.54570 | 1.84680 | 0.19660 | 0.08145 |
| trainlm | poslin-tansig | Training set | 0.91110 | 2.29010 | 5.24440 | 3.35600 | 1.52110 | 0.07875 | 0.06191 |
|  |  | Validation set | 0.85074 | 2.95050 | 8.70550 | 2.71760 | 2.15990 | 0.89898 | 0.14280 |
| trainlm | poslin-logsig | Training set | 0.46896 | 5.75720 | 33.14570 | 1.68710 | 3.83380 | 3.34910 | 0.28665 |
|  |  | Validation set | 0.55926 | 4.61290 | 21.27850 | 1.82120 | 3.13290 | 2.59280 | 0.15573 |
| trainlm | poslin-poslin | Training set | 0.52200 | 5.58090 | 31.14670 | 1.62500 | 3.88660 | 2.54360 | 0.28005 |
|  |  | Validation set | 0.52406 | 4.56330 | 20.82350 | 1.78730 | 3.34540 | 2.66960 | 0.17359 |
| trainlm | poslin-Purelin | Training set | 0.86597 | 2.65680 | 7.05880 | 2.73150 | 1.65070 | 0.01910 | 0.07087 |
|  |  | Validation set | 0.87502 | 3.02070 | 9.12490 | 2.86690 | 2.29850 | 0.49172 | 0.10361 |

**Note:** The dashes indicate invalid entries.
