# Supplementary material for: Mechanism and Prediction of Gray Jujube Fruit Quality Using Explainable ANN
Source: Food Sci Nutr. 2025 Sep 16;13(9):e70928. doi: 10.1002/fsn3.70928 (PMC12438962; doi:10.1002/fsn3.70928)
Supplement: Supplementary file 2 — Appendix S2: fsn370928‐sup‐0002‐AppendixS2.docx. [file FSN3-13-e70928-s002.docx]

**Table 1 Model results of VC model under different seeds**

| Seed | HiddenNodes | RMSE_Modeling | RMSE_Verification | R2_Modeling | R2_Verification | MAE_Modeling | MAE_Verification | MSE_Modeling | MSE_Verification | RPD_Modeling | RPD_Verification | MAPE_Modeling | MAPE_Verification | MBE_Modeling | MBE_Verification |
| --- | --- | --- | --- | --- | --- | --- | --- | --- | --- | --- | --- | --- | --- | --- | --- |
| 42 | 20 | 3.8988 | 7.0694 | 0.99 | 0.95 | 2.8795 | 5.7302 | 15.2005 | 49.9758 | 8.9716 | 4.5925 | 3.7505 | 8.4633 | 0.3759 | 0.6150 |
| 123 | 18 | 2.5180 | 10.5233 | 0.99 | 0.89 | 1.2277 | 6.4499 | 6.3401 | 110.7402 | 13.2378 | 3.5396 | 1.7512 | 8.1180 | 0.1778 | 1.3476 |
| 456 | 18 | 4.9895 | 13.5067 | 0.98 | 0.83 | 1.3745 | 10.0271 | 24.8953 | 182.4301 | 7.0867 | 2.4355 | 2.4213 | 15.2737 | 0.7279 | 0.5312 |
| 789 | 4 | 3.8252 | 8.9003 | 0.99 | 0.92 | 2.5981 | 6.0141 | 14.6318 | 79.2148 | 9.2672 | 3.5582 | 4.0477 | 8.8099 | 0.2398 | 0.8065 |
| 2023 | 4 | 5.2883 | 6.9526 | 0.97 | 0.97 | 3.8511 | 4.9399 | 27.9660 | 48.3390 | 6.0407 | 6.0827 | 0.0492 | 0.0562 | 0.5251 | 2.6131 |
| 1001 | 5 | 4.6342 | 6.7768 | 0.98 | 0.93 | 2.7386 | 4.7008 | 21.4762 | 45.9255 | 8.0056 | 3.9137 | 3.3137 | 6.0511 | 0.3051 | 0.0880 |
| 314159 | 18 | 3.3658 | 7.9411 | 0.99 | 0.93 | 2.2407 | 5.9864 | 11.3284 | 63.0603 | 10.6504 | 3.7339 | 2.8263 | 9.3482 | 0.3515 | 0.9228 |
| 2718 | 14 | 3.1143 | 14.7959 | 0.99 | 0.82 | 0.9728 | 11.0274 | 9.6986 | 218.9179 | 11.0110 | 2.3996 | 1.1070 | 14.0144 | 0.4530 | 1.3043 |
| 1618 | 16 | 6.6450 | 11.0138 | 0.96 | 0.89 | 1.5854 | 7.6678 | 44.1562 | 121.3044 | 5.1851 | 3.0355 | 1.5520 | 13.3551 | 0.3927 | 0.0738 |
| 777 | 18 | 2.9375 | 16.0619 | 0.99 | 0.78 | 1.0070 | 11.1638 | 8.6288 | 257.9831 | 11.7221 | 2.2509 | 1.3093 | 22.1882 | 0.4314 | 5.6113 |

**Table 2 The model results of soluble sugar model under different seeds**

| Seed | HiddenNodes | RMSE_Modeling | RMSE_Verification | R2_Modeling | R2_Verification | MAE_Modeling | MAE_Verification | MSE_Modeling | MSE_Verification | RPD_Modeling | RPD_Verification | MAPE_Modeling | MAPE_Verification | MBE_Modeling | MBE_Verification |
| --- | --- | --- | --- | --- | --- | --- | --- | --- | --- | --- | --- | --- | --- | --- | --- |
| 42 | 19 | 0.4851 | 0.5455 | 0.97 | 0.96 | 0.3154 | 0.4241 | 0.2354 | 0.2976 | 6.1383 | 5.0750 | 4.0956 | 3.4494 | 0.0235 | 0.0964 |
| 123 | 17 | 0.5050 | 0.7259 | 0.97 | 0.95 | 0.3721 | 0.5497 | 0.2550 | 0.5269 | 5.4977 | 4.4343 | 3.3634 | 5.3340 | 0.0298 | 0.1083 |
| 456 | 13 | 0.4760 | 0.7717 | 0.97 | 0.93 | 0.3564 | 0.6022 | 0.2266 | 0.5955 | 6.0083 | 3.8844 | 3.0997 | 8.0716 | 0.0023 | 0.0274 |
| 789 | 18 | 0.3509 | 0.8549 | 0.99 | 0.89 | 0.2433 | 0.5634 | 0.1232 | 0.7309 | 8.3171 | 3.3611 | 2.1592 | 10.8890 | 0.0140 | 0.1019 |
| 2023 | 4 | 0.4698 | 0.6685 | 0.98 | 0.93 | 0.3421 | 0.4816 | 0.2207 | 0.4469 | 6.3593 | 3.8761 | 0.0281 | 0.0748 | 0.0118 | 0.0144 |
| 1001 | 20 | 0.5809 | 1.1654 | 0.97 | 0.45 | 0.1598 | 0.8440 | 0.3375 | 1.3581 | 5.7279 | 1.3493 | 1.1823 | 7.2664 | 0.0657 | 0.0725 |
| 314159 | 12 | 0.3670 | 0.5806 | 0.99 | 0.94 | 0.2658 | 0.3794 | 0.1347 | 0.3371 | 8.3594 | 4.1182 | 2.3457 | 3.6598 | 0.0104 | 0.1056 |
| 2718 | 4 | 0.5027 | 0.7702 | 0.97 | 0.92 | 0.3544 | 0.5496 | 0.2527 | 0.5932 | 5.9577 | 3.5135 | 3.3374 | 4.5448 | 0.0486 | 0.0251 |
| 1618 | 19 | 0.5214 | 0.7659 | 0.97 | 0.93 | 0.3504 | 0.5747 | 0.2719 | 0.5866 | 5.5052 | 3.9052 | 2.9251 | 9.8517 | 0.0091 | 0.2007 |
| 777 | 16 | 0.5523 | 0.7138 | 0.97 | 0.92 | 0.4033 | 0.5257 | 0.3050 | 0.5095 | 5.4380 | 3.6083 | 3.3998 | 4.6279 | 0.0015 | 0.1288 |

**Table 3 Model results of titratable acid model under different seeds**

| Seed | HiddenNodes | RMSE_Modeling | RMSE_Verification | R2_Modeling | R2_Verification | MAE_Modeling | MAE_Verification | MSE_Modeling | MSE_Verification | RPD_Modeling | RPD_Verification | MAPE_Modeling | MAPE_Verification | MBE_Modeling | MBE_Verification |
| --- | --- | --- | --- | --- | --- | --- | --- | --- | --- | --- | --- | --- | --- | --- | --- |
| 42 | 13 | 0.0193 | 0.0449 | 0.94 | 0.65 | 0.0062 | 0.0311 | 0.0004 | 0.0020 | 4.1382 | 1.7045 | 1.2719 | 6.6060 | 0.0001 | 0.0074 |
| 123 | 17 | 0.0208 | 0.0368 | 0.93 | 0.80 | 0.0057 | 0.0261 | 0.0004 | 0.0014 | 3.7135 | 2.2510 | 1.1390 | 5.1558 | 0.0011 | 0.0056 |
| 456 | 11 | 0.0115 | 0.0497 | 0.98 | 0.61 | 0.0071 | 0.0292 | 0.0001 | 0.0025 | 6.8180 | 1.6013 | 1.4510 | 7.2481 | 0.0004 | 0.0015 |
| 789 | 19 | 0.0259 | 0.0330 | 0.90 | 0.80 | 0.0171 | 0.0225 | 0.0007 | 0.0011 | 3.0972 | 2.2652 | 3.4691 | 5.2322 | 0.0002 | 0.0038 |
| 2023 | 4 | 0.0280 | 0.0272 | 0.89 | 0.87 | 0.0208 | 0.0203 | 0.0008 | 0.0007 | 2.8562 | 2.8306 | 0.0431 | 0.0418 | 0.0007 | 0.0071 |
| 1001 | 18 | 0.0273 | 0.0274 | 0.90 | 0.69 | 0.0189 | 0.0219 | 0.0007 | 0.0007 | 3.2259 | 1.7983 | 3.8708 | 4.6873 | 0.0002 | 0.0026 |
| 314159 | 18 | 0.0211 | 0.0336 | 0.93 | 0.73 | 0.0122 | 0.0235 | 0.0004 | 0.0011 | 3.9192 | 1.9104 | 2.4495 | 5.0304 | 0.0009 | 0.0002 |
| 2718 | 14 | 0.0264 | 0.0319 | 0.89 | 0.80 | 0.0177 | 0.0244 | 0.0007 | 0.0010 | 3.0286 | 2.3676 | 3.8017 | 4.8910 | 0.0002 | 0.0042 |
| 1618 | 16 | 0.0274 | 0.0337 | 0.87 | 0.83 | 0.0194 | 0.0216 | 0.0007 | 0.0011 | 2.7907 | 2.4489 | 3.9319 | 5.3502 | 0.0000 | 0.0062 |
| 777 | 15 | 0.0157 | 0.0437 | 0.96 | 0.73 | 0.0095 | 0.0302 | 0.0002 | 0.0019 | 4.7703 | 1.9333 | 1.9290 | 7.2274 | 0.0010 | 0.0017 |

**Table 4 Model results of sugar-acid ratio model under different seeds**

| Seed | HiddenNodes | RMSE_Modeling | RMSE_Verification | R2_Modeling | R2_Verification | MAE_Modeling | MAE_Verification | MSE_Modeling | MSE_Verification | RPD_Modeling | RPD_Verification | MAPE_Modeling | MAPE_Verification | MBE_Modeling | MBE_Verification |
| --- | --- | --- | --- | --- | --- | --- | --- | --- | --- | --- | --- | --- | --- | --- | --- |
| 42 | 14 | 1.4313 | 2.6465 | 0.96 | 0.86 | 1.0664 | 1.8837 | 2.0485 | 7.0040 | 5.2813 | 3.0180 | 4.6371 | 8.7284 | 0.0708 | 0.2592 |
| 123 | 4 | 1.3297 | 2.6443 | 0.97 | 0.87 | 1.0399 | 2.0177 | 1.7681 | 6.9921 | 5.7234 | 3.0885 | 5.0438 | 9.2581 | 0.0783 | 0.7422 |
| 456 | 5 | 1.7329 | 1.9847 | 0.95 | 0.91 | 1.2820 | 1.5192 | 3.0030 | 3.9391 | 4.6770 | 3.3084 | 0.0599 | 0.0629 | 0.0461 | 0.1679 |
| 789 | 13 | 1.3206 | 2.9148 | 0.97 | 0.86 | 0.9666 | 2.0868 | 1.7440 | 8.4963 | 5.7621 | 2.6868 | 4.4828 | 14.7899 | 0.0051 | 0.2275 |
| 2023 | 16 | 1.4302 | 3.3133 | 0.97 | 0.77 | 0.5893 | 2.4616 | 2.0456 | 10.9778 | 5.5075 | 2.0969 | 4.3958 | 12.7946 | 0.1168 | 0.3317 |
| 1001 | 14 | 1.5329 | 2.7207 | 0.97 | 0.68 | 1.1568 | 1.8460 | 2.3499 | 7.4021 | 5.6053 | 1.7686 | 4.9635 | 8.2753 | 0.0583 | 0.0131 |
| 314159 | 20 | 1.1770 | 3.1249 | 0.98 | 0.74 | 0.7493 | 2.3864 | 1.3854 | 9.7647 | 6.9134 | 2.1051 | 3.1853 | 12.5000 | 0.0793 | 1.1583 |
| 2718 | 19 | 1.7150 | 2.0731 | 0.95 | 0.90 | 1.2525 | 1.6328 | 2.9411 | 4.2976 | 4.3682 | 4.0743 | 5.7043 | 6.3899 | 0.0192 | 0.6203 |
| 1618 | 14 | 1.8733 | 1.9515 | 0.94 | 0.93 | 1.4261 | 1.4776 | 3.5093 | 3.8083 | 4.0921 | 3.9134 | 6.1507 | 11.3714 | 0.1813 | 0.3570 |
| 777 | 12 | 1.9743 | 3.9415 | 0.93 | 0.76 | 0.5298 | 3.0197 | 3.8978 | 15.5353 | 3.8179 | 2.1266 | 2.2891 | 22.2303 | 0.3511 | 1.1342 |
